# Quantitative and spatially defined functionalization of nanostructures by overcoming the strong steric hindrance through bioinspired nucleobase interactions

**DOI:** 10.1039/d5sc05777a

**Published:** 2025-10-08

**Authors:** Huijuan Chen, Yuan Xue, Nan Yao, Rong Cheng, Jia Liu, Jiawen Bao, Zan Hua

**Affiliations:** a The Key Laboratory of Functional Molecular Solids, Ministry of Education, Department of Polymer Materials and Engineering, School of Chemistry and Materials Science, Anhui Normal University Wuhu Anhui 241002 China zanhua23@ahnu.edu.cn

## Abstract

Polymer nanostructures with controlled and uniform sizes, tunable surface properties, and spatially defined functionalities are highly pursued by scientists. However, precise control of tailored nanostructures still represents a formidable challenge. Herein, we have successfully developed an efficient and modular route for elegantly functionalizing polymer nanostructures by harnessing robust bioinspired nucleobase interactions of polymers for overcoming the strong steric hindrance. Adenine-containing core-crosslinked nanostructures were rationally designed and fabricated with numerous pendant adenines in the hydrophobic shell layer. Although the adenine-containing hydrophobic shell layer was effectively stabilized by the hydrophilic corona layer, extraordinary multiple hydrogen bonds between bioinspired complementary nucleobase-containing polymers are capable of overcoming the strong steric hindrance for efficient binding. Bioinspired hydrogen bonding interactions of nucleobases display excellent selectivity, superior efficiency, and high association constants. Therefore, it is facile and feasible to tailor the sizes, surface properties, and functionalities of polymer nanostructures through bioinspired nucleobase interactions. Meanwhile, the modular strong interactions of complementary nucleobases enable us to achieve the quantitative core and surface functionalization of synthetic polymer nanostructures. Thus, this work provides a novel and modular strategy for fabricating well-defined nanostructures, which is promising to furnish scalable and elegant nanomaterials for various biological applications such as precise nanovaccines and nanomedicines.

## Abstract 2

Bioinspired nucleobase interactions enable us to elegantly fabricate precise polymer nanostructures with controlled sizes, tunable surface properties, and spatially defined functionalities by overcoming the strong steric hindrance of polymers.

## Graphical abstract

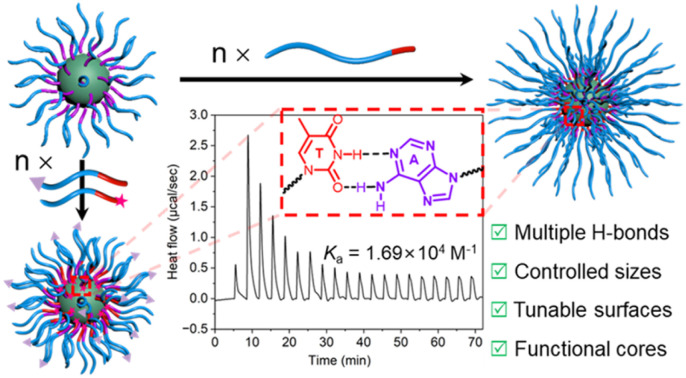

## Introduction

Fabrication of nanostructures with controlled and uniform sizes, tunable surface properties, and spatially defined functionalities is of great significance but still a major challenge.^1–4^ Precise nanostructures are a natural target for nanoscale self-assembly, since they are ubiquitous in biology (*e.g.* in viruses and exosomes) and display extraordinary properties in applications as diverse as nanomedicines,^5,6^ vaccines,^7–9^ and diagnostic tools.^10,11^ DNA nanotechnology enables well-defined nanostructures to be constructed with a high degree of precision, but concerns remain about *in vivo* stability and challenging large-scale synthesis of tailor-made nanomaterials.^12–15^ The formation of elegant DNA nanoparticles mainly relies on the self-assembly of nucleic acids into double helices and the binding to complementary sequences according to programmable Watson–Crick base-pairing. As such, the accessibility of functional handles for further modifications after obtaining nanoparticles is usually limited around the exterior of nanostructures.^16–18^ Recent studies have shown that nucleobase-containing polymers could be harnessed to modulate the properties of nanoparticles *via* intermolecular interactions within the hydrophobic domain.^19–22^ However, the non-crosslinked nature of these nanostructures leads to uncontrolled dissociation upon nucleobase interactions, hindering precise functionalization.

Self-assembly of polymers and living controlled growth of polymer nanoparticles provide two additional approaches for fabricating various nanoscale objects.^23–30^ Polymer solution self-assembly and polymerization induced self-assembly (PISA) are capable of accessing various morphologies such as spheres, worms, and vesicles, but the precise modulation of nanoscale surface properties and functionalities still represents a formidable challenge.^31^ This is mainly due to the uncontrolled fusion or fission of nanoparticles during the process of fabrication. In contrast, the living crystallization-driven self-assembly (CDSA) method has presented an efficient route for constructing micellar assemblies with spatially defined functionalities, which is achieved through seeded growth of crystallizable polymer blocks in the core.^32,33^ However, the epitaxial growth process is usually driven by the formation of crystalline packing between the added unimers and the exposed ends of the seed commonly under heating-annealing processes in non-aqueous media.

The elaborate assembly and complicated functions of nanostructures in biological systems significantly rely on orthogonal supramolecular interactions such as hydrogen bonds and hydrophobic interactions.^34^ For example, at the nanoscale of cells, different nanostructures consisting of dynamic amphiphilic lipid bilayers are able to combine and fuse for exerting biological functions. Based on a similar mechanism, scientists have developed numerous lipid-based nanocarriers, showcasing remarkable properties and superior efficacy.^35–37^ In stark contrast, it is usually difficult for polymer self-assemblies with amphiphilic blocks to achieve similar controlled processes owing to the strong steric hindrance of dense hydrophilic chains and kinetically frozen cores.^38,39^ The lack of driving forces makes the transformation or functionalization processes thermodynamically unfavorable, hindering selective and quantitative modifications of nanostructures for various applications. Therefore, it is highly desired to develop a straightforward and robust approach for precisely sculpting nanostructures.

Herein, we have developed a straightforward and efficient method for tailoring the structures and properties of polymer nanostructures, by leveraging strong and specific multiple hydrogen bonds between complementary nucleobase-containing polymers. A series of stable adenine-containing core-crosslinked nanostructures were fabricated through the fast photocrosslinking of coumarins while maintaining pendant adenines intact for functionalization. Although the adenine-containing hydrophobic shell layer was stabilized by the hydrophilic corona layer, robust bioinspired multiple hydrogen bonds between complementary nucleobase-containing polymers are strong enough to overcome the strong steric hindrance for binding. Thymine-containing polymers with hydrophilic chains of different lengths all exhibited nearly quantitative bonding to the adenine-containing core-crosslinked nanostructure. As such, polymer nanostructures with distinct sizes, surface properties, and functionalities were successfully fabricated by employing robust bioinspired nucleobase interactions. Furthermore, it is also feasible to simultaneously implement quantitative core and surface functionalization of polymer nanostructures. Altogether, bioinspired nucleobase interactions in the hydrophobic domain significantly expand available handles for effectively modulating polymer nanostructures, which is poised to produce scalable and precise nanomaterials especially for various biological applications.

## Results and discussion

### Construction of nucleobase-containing core-crosslinked nanostructures

**Fig. 1:**
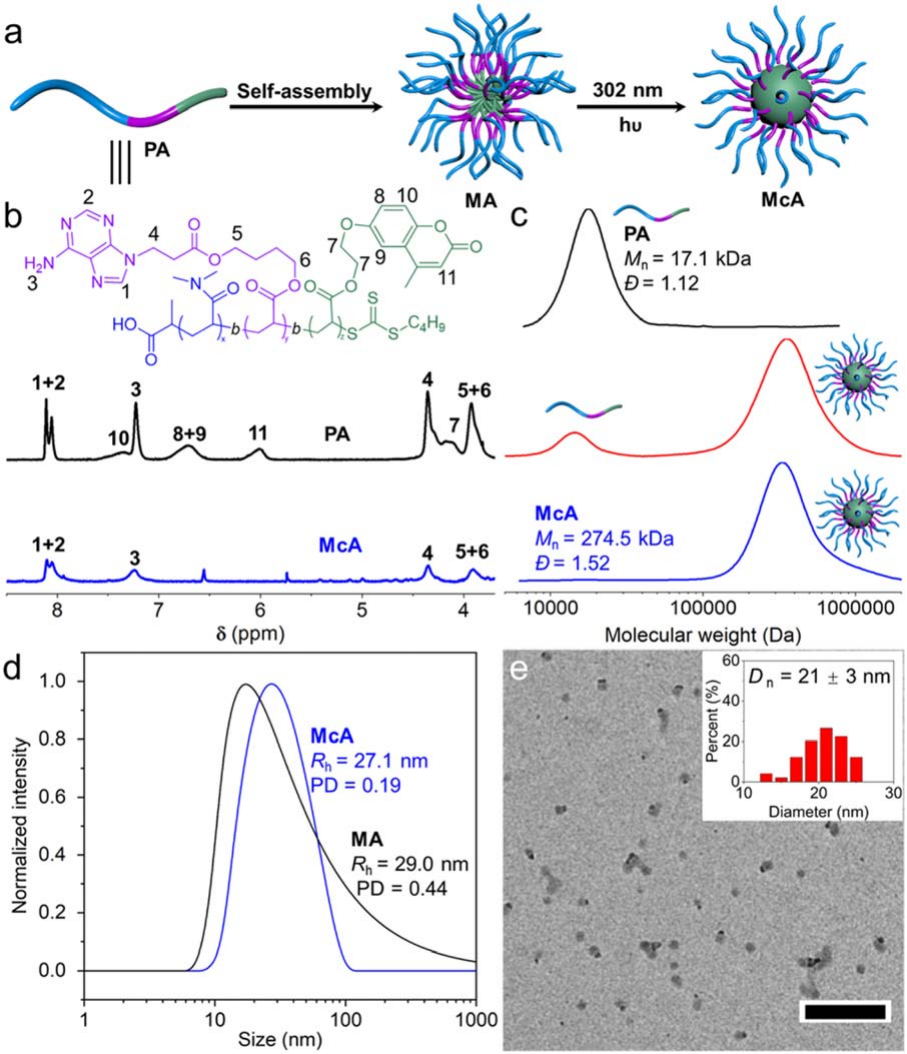
Characterization of the adenine-containing crosslinked polymer and nanostructures. (a) Schematic illustration of synthesis of the adenine-containing polymer and core-crosslinked nanostructure. (b) ^1^H NMR spectra and (c) DMF SEC traces of the adenine-containing polymers before and after crosslinking. (d) DLS analyses of the adenine-containing nanostructures and (e) TEM micrograph of the adenine-containing core-crosslinked nanostructure McA, with the inset showing the size distribution. Scale bar: 200 nm.

Nucleobase-containing triblock copolymers poly(*N*,*N*-dimethylacrylamide)-*b*-poly(4-((3-(adenine-9-yl)propanoyl)oxy)butyl acrylate)-*b*-poly(2-((4-methylcoumarin-7-yl)oxy)ethyl acrylate)s (PDMA-*b*-PAAc-*b*-PMCAcs) were prepared through sequential RAFT polymerizations (Fig. S1–S7). The attained triblock copolymer PDMA_40_-*b*-PAAc_20_-*b*-PMCAc_20_ (PA) was able to self-assemble into the core-shell-corona nanostructure MA, which could be UV-irradiated to afford the adenine-containing core-crosslinked nanostructure McA (Fig. 1a). In the ^1^H NMR spectra the peaks at 7.35, 6.72, and 6.02 ppm assigned to coumarin were observed to almost disappear (Fig. 1b). Meanwhile, the peaks at 8.11 and 7.24 ppm for adenine were almost retained, suggesting the intactness of the adenine functionality in the crosslinked nanostructure. The crosslinking kinetics of MA was monitored by using UV-vis spectroscopy and SEC analyses. A fast decrease in UV absorbance at 319 nm was first observed in the initial 15 min, followed by a slow decrease upon further UV irradiation (Fig. S8). It should be noted that no obvious crosslinking occurs for PDMA_40_-*b*-PAAc_20_ due to the lack of UV-reactive groups (Fig. S9). SEC traces manifested that a new polymer peak with a number-average molar mass *M*_n_ and molar mass dispersity *Ɖ* of 226.5 kDa and 1.20 initially emerged after 30 min of UV irradiation (Fig. S10), illustrating the formation of crosslinked polymers. The weight ratio of crosslinked polymers was over 86% after 4 h and no further increase was observed when extending the time of UV irradiation.

In order to avoid the influence of uncrosslinked polymers, the core-crosslinked polymers were purified and separated by using preparative Size Exclusion Chromatography (SEC). As shown in Fig. 1c, only adenine-containing crosslinked polymers with an *M*_n_ and *Ɖ* of 274.5 kDa and 1.52 were observed after removing the uncrosslinked polymers. The obtained adenine-containing core-crosslinked polymers could afford the well-defined nanostructure McA, which was characterized by using DLS and TEM imaging. DLS analysis showed that McA had a hydrodynamic radius and polydispersity of 27.1 nm and 0.19 (Fig. 1d), both presenting a decrease in comparison to MA. In addition, the TEM image also indicated that the nanoparticles McA with a diameter of 21 ± 3 nm were generated (Fig. 1e). Based on the robust and efficient approach, a series of stable adenine-containing nanostructures with different sizes can be constructed by changing the block lengths for the crosslinked cores (Fig. S11–S15). The core-crosslinking of PA is more efficient for the formation of crosslinked nanoparticles compared to PDMA_40_-*b*-PAAc_20_-*b*-PMCAcs with the DPs of PMCAc of 10 or 30. On this basis, McA from PA should be further investigated for functionalization modifications. Collectively, we have developed an avenue for successfully fabricating adenine-containing core-crosslinked nanostructures.

### Complementary nucleobase interactions for core-crosslinked nanostructures

Post-modifications of amphiphilic polymeric nanoparticles were previously achieved mainly through the corona layer. In contrast, the hydrophobic layer was rarely employed for nanoparticle functionalization due to both the strong steric hindrance of the hydrophilic layer and the low accessibility of the functional groups in the hydrophobic layer. The core-crosslinked nanostructure McA contains numerous pendant adenines in the shell layer, which was stabilized by the hydrophilic PDMA. We wonder whether the robust bioinspired hydrogen bonds of complementary nucleobases can overcome the strong steric hindrance for achieving quantitative functionalization of nanostructures. Thymine-containing polymers poly(*N*,*N*-dimethylacrylamide)-*b*-poly(4-((3-(thymin-1-yl)propanoyl)oxy)butyl acrylate) (PDMA_40_-*b*-PTAc_20_ (PT1), PDMA_100_-*b*-PTAc_20_ (PT2), and PDMA_200_-*b*-PTAc_20_ (PT3)) were prepared with the thymine-containing block of the same length but varied PDMA blocks (Fig. S16–S18). All thymine-containing polymers PT1–PT3 yield dynamic micelles MT1–MT3 after direct dissolution in water.

**Fig. 2:**
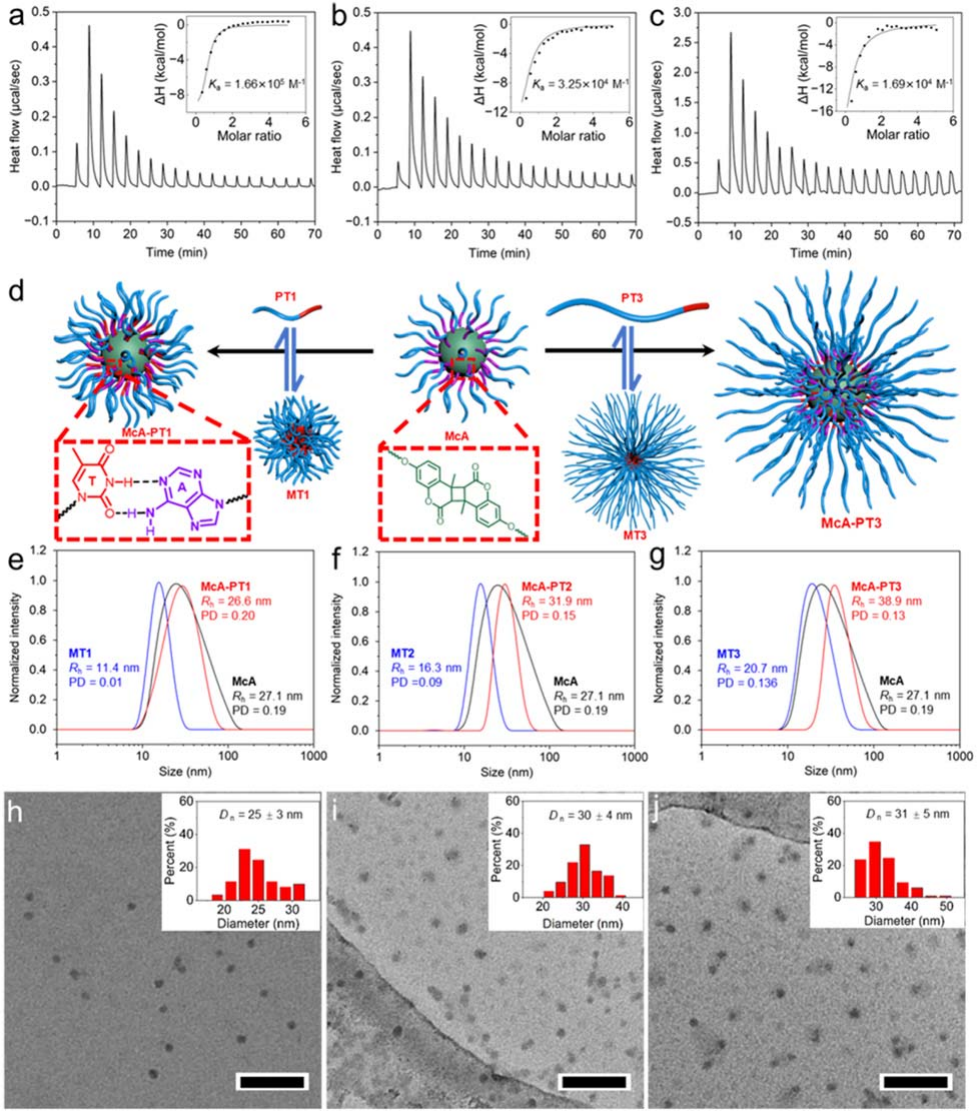
Interactions between McA and MTs through complementary nucleobases. ITC analyses of the interaction between McA with (a) MT1, (b) MT2, and (c) MT3. (d) Schematic of the formation of combined nanostructures. DLS analyses of (e) McA-PT1, (f) McA-PT2, and (g) McA-PT3 in comparison with McA and MTs. TEM micrographs of (h) McA-PT1, (i) McA-PT2, and (j) McA-PT3, with the insets showing the size distribution. Scale bar: 200 nm.

Initially, the interactions between McA and MT1–MT3 were investigated by using Isothermal Titration Calorimetry (ITC). As shown in Fig. 2a–c, obvious heat release was observed and high association constants *K*_a_s were obtained, ranging from 1.66 × 10^5^ M^−1^ for MT1 to 1.69 × 10^4^ M^−1^ for MT3. The high *K*_a_ values indicate that most of the added MTs were combined with McA. As expected, the larger molecular sizes of PT2 and PT3 for MT2 and MT3 give rise to higher steric hindrance for binding McA, resulting in lower binding constants. To explore the importance of hydrogen bonds of complementary nucleobases, we further study the interaction between McA and PDMA_40_-*b*-PAAc_20_ (PA0) or poly(*N*,*N*-dimethylacrylamide)-*b*-poly(4-((3-(3-methylthymin-1-yl)propanoyl)oxy)butyl acrylate) (PDMA_40_-*b*-PMTAc_20_, PMT) (Fig. S19 and S20). PA0 and PMT contain either non-complementary nucleobase adenine or methylated thymine, both of which lack the selective and specific hydrogen bonds with McA. Interestingly, no discernible binding to McA was observed after adding self-assemblies of either PA0 or PMT (Fig. S20), illustrating that hydrophobic interaction and π–π stacking in the hydrophobic domain are not strong enough to drive the functionalization of McA.

On this basis, we propose that the efficient combination between McA and MTs for the modification of nanostructures is attributed to the strong hydrogen bonds between complementary nucleobases adenine and thymine (Fig. 2d). The high combination enthalpy due to the interaction of adenine and thymine-containing polymers is enough to compensate for the entropic penalty of the stretching of shell and corona layers, leading to the spontaneous combination between MT and McA. Furthermore, DLS analyses were employed to interrogate the efficient binding between MTs and McA (Fig. 2e–g). As PT1 has the same chain length for the PDMA block compared with McA, the association is not able to increase the size of the nanostructure significantly. For both PT2 and PT3 with longer hydrophilic blocks, the combined nanostructures McA-PT2 and McA-PT3 have the hydrodynamic radii of 31.9 and 38.9 nm, increasing from that of McA of 27.1 nm. Notably, the covalent core-crosslinked structure is of great significance for the formation of combined nanostructures without disassembly. Conversely, the mixing of non-crosslinked micelles of adenine and thymine-containing diblock copolymers only leads to a slight decrease in the sizes compared with constituent nanostructures (Fig. S21). The combined nanostructures were also analyzed by using TEM imaging (Fig. 2h–j). Larger nanoparticles with a slightly blurry surface were observed, confirming the formation of combined nanostructures through bioinspired nucleobase interactions. Therefore, multiple hydrogen bonds of nucleobases enable us to elegantly modulate the sizes of nanostructures by overcoming the strong steric hindrance of polymers.

### Precise surface functionalization of nucleobase-containing nanostructures

**Fig. 3:**
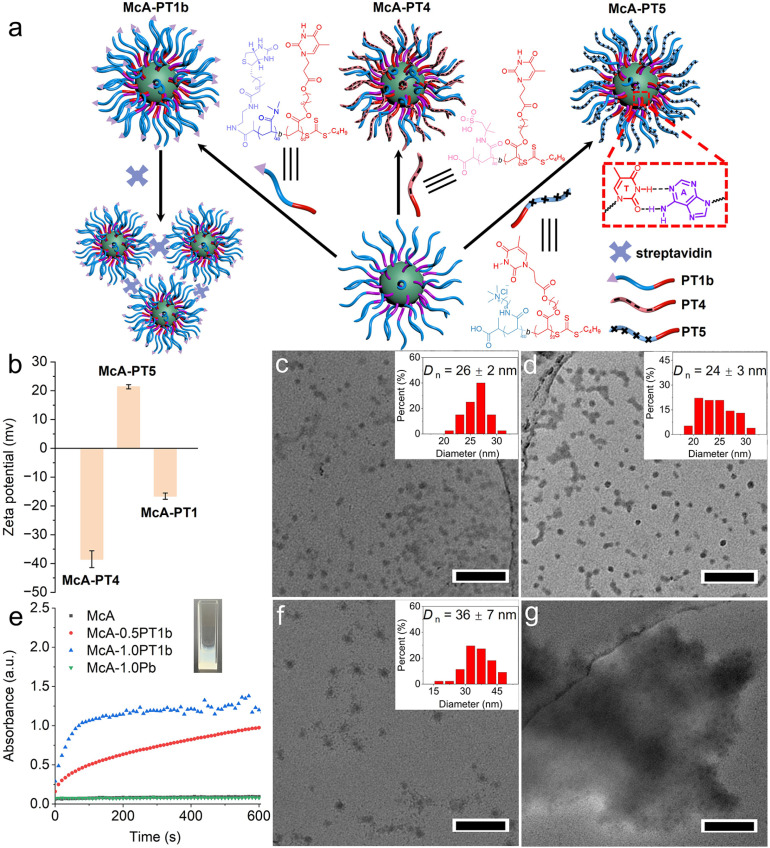
Surface functionalization of nucleobase-containing nanostructures. (a) Schematic illustration of surface modifications of McA through complementary nucleobases. (b) Zeta potentials and (c and d) TEM micrographs of McA-PT4 and McA-PT5 modified with cationic or anionic polymers. (e) The aggregation process after adding streptavidin into biotin-functionalized nanostructures McA-*x*PT1b and control samples monitored by UV-vis spectroscopy, with the inset showing the solution after the addition of streptavidin into McA-1.0 PT1b. TEM micrographs of McA-1.0 PT1b before (f) and after (g) the addition of streptavidin. The insets of (c), (d), and (f) indicate the size distribution of nanoparticles. Scale bar: 200 nm.

Robust nucleobase interactions provide a superb and modular method for tailoring surface properties of nanostructures *via* a “grafting to” approach (Fig. 3a). As shown in Fig. 3b–d and S22, S23, well-defined spherical nanoparticles McA-PT4 and McA-PT5 with distinct zeta potentials were yielded after adding poly(2-acrylamido-2-methyl-1-propanesulfonic acid)-*b*-poly(4-((3-(thymin-1-yl)propanoyl)oxy)butyl acrylate) (PAMPS_40_-*b*-PTAc_20_, PT4) or poly(3-(acrylamido)propyltrimethylammonium)-*b*-poly(4-((3-(thymin-1-yl)propanoyl)oxy)butyl acrylate) (PTMPA_40_-*b*-PTAc_20_, PT5). The introduction of anionic polymers PAMPS on the surface afforded McA-PT4 with a zeta potential of −38.5 ± 2.9 mV. It should be noted that McA-PT1 also has a negative zeta potential of −16.6 ± 1.1 mV, which is due to the carboxylate group at the hydrophilic end of polymers. Interestingly, McA-PT5 presented a positive zeta potential of 21.4 ± 0.7 mV due to the binding of the cationic polymer PT5. Meanwhile, TEM images show that both McA-PT4 and McA-PT5 display similar diameters of *ca.* 25 nm (Fig. 3c and d). These results suggest that bioinspired nucleobase interactions are efficient to modulate the charged properties of nanoparticles while maintaining the same sizes.

To further explore the precise and quantitative surface functionalization of nanostructures, biotin-attached PDMA_40_-*b*-PTAc_20_ (PT1b) was prepared to interact with McA through bioinspired nucleobase interactions (Fig. S24 and S25). The surface functionalized nanostructures McA-*x*PT1b were fabricated, in which *x* represents the molar ratio of thymine to adenine (Fig. 3a). The aggregation of biotin-modified nanoparticles was investigated by adding streptavidin, which selectively binds four biotin molecules for precipitation. The aggregation process was monitored by using UV-vis spectroscopy at 500 nm. The solutions of McA-1.0 PT1b and McA-0.5 PT1b quickly turned cloudy after the introduction of streptavidin (Fig. 3e). Higher surface densities of biotins for McA-1.0 PT1b result in faster aggregation in comparison to McA-0.5 PT1b. In contrast, no discernible aggregation was detected for either McA or the mixture (McA-1.0Pb) of McA and biotin-attached PDMA_40_ (Pb). The aggregates after adding streptavidin into McA-1.0 PT1b were centrifuged and characterized by TEM imaging. As shown in Fig. 3f and g, large aggregates consisting of small nanoparticles (about 30 nm) were clearly observed, confirming the efficient aggregation of McA-1.0 PT1b by streptavidin. Thus, the precise and efficient surface functionalization of nanostructures can be achieved by utilizing complementary nucleobase interactions in the hydrophobic domain.

### Quantitative core and surface modifications of nucleobase-containing nanostructures

**Fig. 4:**
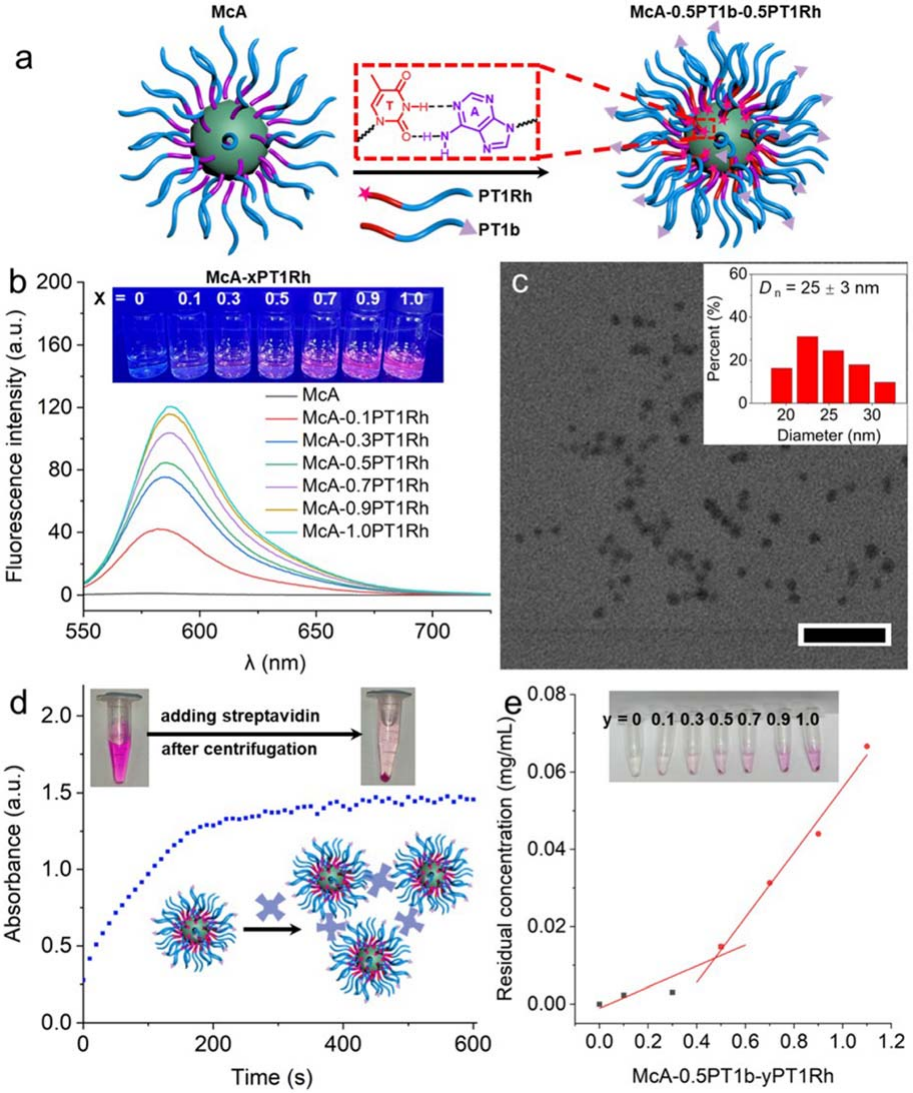
Core and surface functionalization of nucleobase-containing nanostructures. (a) Schematic illustration of quantitative core and surface modifications of McA. (b) Fluorescence spectra of McA-*y*PT1Rh with the introduction of varying quantities of PT1Rh with the insets showing the digital photos under UV illumination. (c) TEM micrograph of McA-1.0PT1Rh with the inset showing the size distribution. Scale bar: 200 nm. (d) The aggregation process of McA-0.5PT1b-0.5PT1Rh monitored by UV-vis spectroscopy. (e) The residual PT1Rh dependence on McA-0.5PT1b-*y*PT1Rh after adding streptavidin. The insets in (d) and (e) show the samples upon the addition of streptavidin.

The adenine-containing hydrophobic layer of nanostructures lies in the hydrophobic shell between the crosslinked core and the hydrophilic corona, which also enables us to functionalize the hydrophobic domain (Fig. 4a). Rhodamine-attached PDMA_40_-*b*-PTAc_20_ (PT1Rh) with a fluorescent group at the hydrophobic end was synthesized (Fig. S26–S28). A series of fluorescent nanostructures McA-*y*PT1Rh were fabricated by modulating the added quantities of PT1Rh into McA, in which *y* represents the molar ratio of thymine to adenine. The fluorescence intensity presents a gradual increase once more PT1Rh molecules were included in the combined nanostructures (Fig. 4b). As shown in Fig. 4c, TEM imaging further verified that well-defined spherical nanoparticles were generated for McA-1.0 PT1Rh. As a result, it is straightforward and feasible to realize quantitative core modifications of nucleobase-functionalized nanostructures for fluorescent properties.

Based on the modular functionalization of nucleobase-containing nanostructures, the simultaneous core and surface modifications were undertaken by introducing PT1Rh and PT1b into McA. Obvious precipitates were yielded after adding streptavidin into the McA-0.5PT1b-0.5 PT1Rh solution (Fig. 4d). Red aggregates were collected at the bottom after centrifugation and the solution is light red, meaning the successful core and surface functionalization of McA. In order to quantify the core functionalization, different molar ratios of PT1Rh were utilized for functionalizing McA-0.5PT1b to deliver McA-0.5PT1b-*y*PT1Rh. The excess PT1Rh would remain in the supernatant after the aggregation of McA-0.5PT1b-*y*PT1Rh triggered by streptavidin. As shown in Fig. 4e, UV-vis spectra were employed to monitor the supernatant, suggesting the critical point of McA-0.5PT1b-0.5 PT1Rh with the saturation of adenine-thymine interactions. These results illustrate that adenine-containing core-crosslinked nanostructures can be functionalized quantitatively considering the strong binding between polymers with bioinspired complementary nucleobases.

## Conclusion

In summary, we have reported an efficient and modular avenue for realizing precise and quantitative functionalization of nanostructures by harnessing bioinspired nucleobase interactions in the hydrophobic domain. Specifically, adenine-containing core-crosslinked nanostructures were constructed, which had numerous pendant adenines in the hydrophobic shell layer and were stabilized by the hydrophilic polymer as the corona. Strong and selective multiple hydrogen bonds between complementary nucleobases were found to be capable of overcoming the strong steric hindrance between polymers for the nanostructure modifications, manifesting superior efficiency and high association constants. Depending on the robust bioinspired nucleobase interactions, it is straightforward and feasible to tailor the sizes, surface properties, and functionalities of nanostructures. Importantly, the modular strong interactions of complementary nucleobases make it possible to implement quantitative core and surface functionalization of synthetic polymer nanostructures. Therefore, this work provides an interesting and novel strategy for fabricating precise nanostructures, which are promising to unravel the structure–function relationship and amplify the biological applications of polymer nanoparticles.

## Author contributions

The manuscript was written through contributions of all authors. All authors have given approval to the final version of the manuscript. Z. H. and H. C. conceived and designed the study. H. C. carried out most of the experiments and analysed the related data. Y. X., N. Y., R. C., J. L., and J. B. participated in the experiments for monomer and polymer syntheses.

## Conflicts of interest

The authors declare no competing financial interest.

## Supplementary Material

SC-OLF-D5SC05777A-s001

## Data Availability

The data that support the findings of this study are available in the supporting information (SI) of this article. Supplementary information: experimental details, ^1^H NMR spectra, size exclusion chromatography, UV-vis spectra, fluorescence spectra, dynamic light scattering, transmission electron microscopy and isothermal titration calorimetry of nucleobase-containing polymers (PDF). See DOI: https://doi.org/10.1039/d5sc05777a.
