# Supplementary material for: Quantitative and spatially defined functionalization of nanostructures by overcoming the strong steric hindrance through bioinspired nucleobase interactions
Source: Chem Sci. 2025 Oct 8;16(43):20594–600. doi: 10.1039/d5sc05777a (PMC12506782; doi:10.1039/d5sc05777a)
Supplement: SC-016-D5SC05777A-s001 [file SC-016-D5SC05777A-s001.pdf]

Supplementary Information (SI) for Chemical Science.
This journal is © The Royal Society of Chemistry 2025

Supporting Information

Quantitative and Spatially Defined Functionalization of Nanostructures by Overcoming the Strong Steric Hindrance through Bioinspired Nucleobase Interactions

Huijuan Chen,[a] Yuan Xue,[a] Nan Yao,[a] Rong Cheng,[a] Jia Liu,[a] Jiawen Bao,[a] and Zan Hua[*a]*

[a]The Key Laboratory of Functional Molecular Solids, Ministry of Education, and Department of Polymer Materials and Engineering, School of Chemistry and Materials Science, Anhui Normal University, Wuhu, Anhui, 241002, China. E-mail: zanhua23@ahnu.edu.cn

Contents

Materials and methods 3

Materials 3

Methods 4

Synthetic procedures 5

Synthesis of 4-((3-(methylthymin-1-yl)-propanoyl)oxy)butyl acrylate (MTAc) 5

Synthesis of biotin-CTA 6

Synthesis of 3-((12-(2-(((butylthio)carbonothioyl)thio)propanamido)dodecyl)-carbamoyl)-4-(6-(dimethylamino)-3-(dimethyliminio)-3H-xanthen-9-yl)benzoate (Rh-CTA) 7

Syntheses of PDMA, PDMA-*b*-PAAc$_{20}$, PDMA-*b*-PTAc$_{20}$ (PT), PDMA-*b*-PMTAc$_{20}$ (PMT) 9

Synthesis of PDMA$_{40}$-*b*-PAAc$_{20}$-*b*-PMCAcs 11

Synthesis of PAMPS$_{40}$-*b*-PTAc$_{20}$ (PT4) and PTMPA$_{40}$-*b*-PTAc$_{20}$ (PT5) 12

Synthesis of biotin-attached PDMA$_{40}$-*b*-PTAc$_{20}$ (PT1b) and rhodamine-modified PDMA$_{40}$-*b*-PTAc$_{20}$ (PT1Rh) 14

Crosslinking and purification of self-assembled polymer nanoparticles 16

Interactions between McA nanostructure and MTs 16

Supplementary Figures 17

References 26

Materials and methods

Materials

N-Hydroxysuccinimide (NHS, 99%, Maclin), 4-dimethylaminopyridine (DMAP, 99%, Macklin), *N*,*N*'-dicyclohexylcarbodiimide (DCC, 99%, Aladdin), *N*,*N*-diisopropylethylamine (DIPEA, 99%, Aladdin), dichloromethane (DCM, 99.5%, Sinopharm), *N*,*N*-dimethylformamide (DMF, 99.5%, Sinopharm), 2-acrylamido-2-methyl-1-propanesulfonic acid (AMPS, AR, Aladdin), 3-(acrylamido)propyltrimethylammonium chloride (TMPA, 74-76 wt% in water, Heowns), biotinylamidoethylacetamide (biotin-NH_2, 95%, Meryer), streptavidin (≥12 U/mg protein, Aladdin), 3-carboxy-4-(6-(dimethylamino)-3-(dimethyliminio)-3H-xanthen-9-yl)benzoate (Rh-COOH, 98%, Macklin), *tert*-butyl(6-aminohexyl)carbamate (97%, Meryer), *N*-(3-dimethylaminopropyl)-*N*'-ethylcarbodiimide hydrochloride (EDC.HCl, 99%, Bidepharm), and 1-hydroxybenzotriazole (HOBt, ≥97%, Macklin) were purchased and used directly without further purifications. 2-(((Butylthio)carbonothiolyl)thio)propanoic acid (CTA), 2,5-dioxopyrrolidin-1-yl-2-(((butylthio)carbonothioyl)thio)pro-panoate (CTA-NHS), 4-((3-(thymin-l-yl)propanoyl)oxy)butyl acrylate (TAc), 4-((3-(adenin-9-yl)propanoyl)oxy)butyl acrylate (AAc), and 2-((4-methylcoumarin-7-yl)oxy)ethyl acrylate (MCAc) were prepared as reported previously (Figures S1-S4).[1-4]

Methods

All ^{1}H, ^{13}C NMR spectra were recorded on a 400/500 MHz spectrometer (AVANCE400, Bruker, USA) with $CDCl_3$ or DMSO-d_6 as the solvent by using tetramethylsilane (TMS) as the internal standard or referring to the solvent residual peaks. The number-average molecular mass (M_n) and molecular mass distribution ($Đ$) of polymers were measured by using Size Exclusion Chromatography (SEC, Agilent 1260), which was equipped with a PLgel column using DMF (containing 5 mM NH_4BF_4) as the eluent with a flow rate of 1 mL min^{-1} at 50 °C. Poly(methyl methacrylate) standards were used as the calibration polymers prior to use. Samples were filtered through a nylon filter (pore size of 0.22 µm) prior to SEC characterization. The core-crosslinked polymer nanoparticles were purified and separated by using preparative Size Exclusion Chromatography (SEC, SHIMADZU), which was equipped with a PLgel column using DMF (containing 5 mM NH_4BF_4) as the eluent with a flow rate of 5 mL min^{-1} at 50 °C. The dynamic light scattering (DLS) instrument (ALV/CGS-8F, ALV, German) was used for particle size and particle distribution analyses at 25 °C. The crosslinking kinetics of MCA upon UV irradiation were monitored by using SEC and UV-vis spectrophotometer (FC-3100, Life Real, China). Transmission electron microscope (TEM) imaging (HT-7700 microscope, Hitachi, Japan) was carried out under vacuum to observe the morphologies of the nanostructures. The sample solution of 5 µL was dropped on a hydrophilic ultra-thin carbon layer for 1 min, then blotted away. The sample without staining was further dried at room temperature and characterized by TEM. The average sizes of nanoparticles were analyzed and calculated by counting at least over 100 particles

using ImageJ software. UVP Crosslinker (analytikjena) was used to afford the adenine-containing core-crosslinked nanostructures. The fluorescence intensity was recorded by fluorescence spectrophotometer (Hitachi, F-4700, Japan). The interaction between nucleobase-containing polymers was measured by isothermal titration calorimetry (ITC, ITC200, TA Instruments, USA).

Synthetic procedures

Synthesis of 4-((3-(methylthymin-1-yl)-propanoyl)oxy)butyl acrylate (MTAc)

A mixture of 4-((3-(thymin-l-yl)propanoyl)oxy)butyl acrylate (TAc) (973 mg, 3 mmol), dry K_2CO_3 (660 mg, 4.8 mmol), and iodomethane (765 µL, 98%) in anhydrous DMF (4 mL) was stirred at room temperature for 24 h (Scheme S1). Then the mixture was diluted with ethyl acetate (50 mL), washed with water (2 × 50 mL), and dried with anhydrous Na_2SO_4. The solvent was removed under vacuum and the obtained monomer MTAc (711 mg, 70.0% yield) was dried in a vacuum oven overnight at room temperature and analyzed by ^{1}H and ^{13}C NMR spectroscopy (Figure S2). ^{1}H NMR (400 MHz, DMSO-d_6): δ = 1.58−1.68 (m, 4H, -O-CH_2-CH_2-CH_2-CH_2-O-), 1.79 (s, 3H, CH_3-pyrimidine), 2.71 (t, 2H, -O(C=O)-CH_2-), 3.16 (s, 3H, -(C=O)-N-CH_3), 3.91 (t, 2H, CH_2-pyrimidine), 4.00−4.15 (m, 4H, -O-CH_2-CH_2-CH_2-CH_2-O-), 5.95 (dd, 1H, -CH_2=CH-), 6.17 (dd, 1H, -CH_2=CH-(C=O)-), 6.29 (dd, 1H, -CH_2=CH-), 7.57 (s, 1H, pyrimidine=CH). ^{13}C NMR (125 MHz, DMSO-d_6): δ = 170.8, 165.5, 163.3, 151.0, 140.2, 131.5, 128.3, 107.3, 63.7, 44.9, 32.7, 27.4, 24.7, 12.6 ppm; HR-MS (m/z) found 339.1555, calcd 339.1551

[M + H]$^+$.

Scheme S1. Synthesis of 4-((3-(methylthymin-l-yl)propanoyl)oxy)butyl acrylate (MTAc).

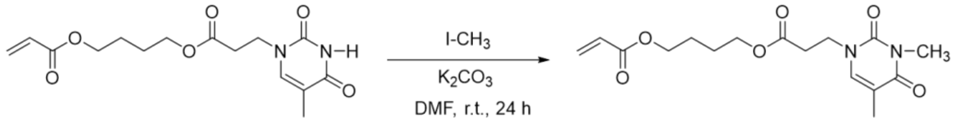

Synthesis of biotin-CTA

The details of the procedures are as follows. CTA-NHS (436 mg, 1.30 mmol) and *N*, *N*-diisopropylethylamine (DIPEA) (280 μL, 1.56 mmol) were first dissolved in DMF. Then, biotinylamidoethylacetamide (biotin-NH_2) (372 mg, 1.30 mmol) was added into the mixture and stirred overnight at room temperature (Scheme S2). The solvent was removed after the reaction. The mixture was further purified *via* column chromatography with a mixture of CH_2Cl_2/CH_3OH (v:v 9:1) to give a yellow solid (430 mg, 65.3% yield). The assigned ^{1}H NMR spectra were shown in Figure S24. ^{1}H NMR (400 MHz, DMSO-d_6) : δ = 0.88 (t, 3H, CH_3-CH_2-), 1.21-1.52 (m, 10H), 1.56-1.66 (m, 2H, -S-CH_2-CH_2-CH_2-), 2.0-2.1 (t, 2H, -CH_2-CH_2-(C=O)-), 2.55-2.86 (m, 2H, -CH-CH_2-S-), 3.02-3.16 (m, 4H, -NH-CH_2CH_2-NH-), 3.34-3.36 (m, 2H, -S-CH_2-CH_2-), 3.54–3.67 (m, 1H, -CH_2-C*H*-S-), 4.08-4.18 (m, 1H, -NH-C*H*-CH_2-), 4.25-4.36 (m, 1H, -NH-C*H*-CH-), 4.60-4.73 (m, 1H, -CH_3-C*H*-(C=O)-), 6.28-6.55 (d, 2H, -(C=O)-N*H*-CH-), 7.71-7.98 (s, 1H, -CH_2-N*H*-(C=O)-), 8.29-8.49 (s, 1H, -(C=O)-N*H*-CH_2-). ^{13}C NMR (100 MHz, DMSO-d_6): δ = 223.0, 172.2, 169.4, 162.7, 61.0, 59.2, 55.4, 53.5, 50.0, 38.0, 36.1, 35.3, 29.6, 28.2, 25.2, 21.4, 18.2, 16.7, 13.4, 12.3 ppm; HR-MS (m/z) found 507.1602, calcd 507.1587 [M + H]$^+$.

Scheme S2. Synthesis of biotin-CTA.

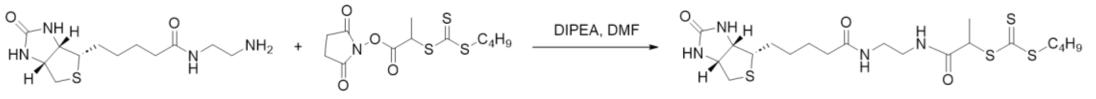

Synthesis of 3-((12-(2-(((butylthio)carbonothioyl)thio)propanamido)dodecyl)-carbamoyl)-4-(6-(dimethylamino)-3-(dimethyliminio)-3H-xanthen-9-yl)benzoate (Rh-CTA)

The synthesis of Rh-CTA was carried out through the reaction of carboxyl acid of rhodamine and the amidation reaction sequentially (Scheme S3). 3-Carboxy-4-(6-(dimethylamino)-3-(dimethyl-liminio)-3H-xanthen-9-yl)benzoate (Rh-COOH) (258 mg, 0.6 mmol) was dissolved in DMF, and then *tert*-butyl(6-aminohexyl)carbamate (184.8 mg, 0.92 mmol), *N*-(3-dimethylaminopropyl)-*N'*-ethylcarbodiimide hydrochloride (EDC.HCl) (223 mg, 1.16 mmol), 1-hydroxybenzotriazole (HOBt) (157 mg, 1.16 mmol), and *N*,*N*-diisopropylethylamine (DIPEA) (204 µL, 1.16 mmol) were added sequentially. The solution was stirred overnight at room temperature. The mixture was poured into dichloromethane and the organic layer was washed by water (3 × 300 mL). Then the organic layer was dried over anhydrous $MgSO_4$, filtered and concentrated via vacuum evaporation to remove the solvent. The evaporation residue was further purified using flash chromatography with the mixture of CH_2Cl_2 and MeOH (v:v 6:1) as eluent to yield Rh-BocNH$_2$ (Figure S26). ^{1}H NMR (400 MHz, DMSO-d_6): δ = 1.16-1.61 (m, 17H, -C-CH_3, -CH$_2$-CH_2-CH_2-CH_2-CH_2-CH$_2$-), 2.92-2.99 (s, 12H, -N-CH_3, -N$^+$-CH_3), 3.22-3.35 (m, 2H, -NH-CH_2-CH$_2$-), 3.35-3.65 (m, 2H, -CH$_2$-CH_2-NH-), 6.43-6.55 (m, 6H, benzene-*H*), 6.70-

6.82 (s, 1H, -CH_2-N*H*-(C=O)-), 7.24-8.47 (m, 3H, benzene-*H*), 8.76-8.86 (s, 1H, -(C=O)-N*H*-CH_2-). ^{13}C NMR (100 MHz, DMSO-d_6): δ = 168.6, 164.9, 155.9, 154.8, 152.3, 152.2, 136.4, 134.6, 128.6, 127.1, 124.3, 123.3, 109.2, 105.7, 98.1, 85.3, 77.6, 29.6, 29.1, 28.4, 26.4, 26.2 ppm; HR-MS (m/z) found 629.3338, calcd 629.3334 $[M + H]^+$.

For the synthesis of Rh-CTA, the attained Rh-BocNH_2 (327 mg, 0.52 mmol) was deprotected in the mixture of trifluoroacetic acid (TFA) and DCM (v:v 3:1) for 12 h. The mixture was concentrated to yield Rh-NH_2. Then, CTA-NHS (348.4 mg, 1.04 mmol) and DIPEA (362 μL, 2.08 mmol) were dissolved in DMF (1 mL). Rh-NH_2 (274 mg, 0.52 mmol) was slowly added into the mixed solution and stirred overnight at room temperature (Scheme S3). The residue was partitioned with DCM and water. The aqueous layer was extracted three times with DCM and the combined organic layers were dried over anhydrous Na_2SO_4. The residue was purified by column chromatography with a gradient of DCM/CH_3OH (10:0 to 10:1) to get the product (216 mg, 55.4% yield) (Figure S27). ^1H NMR (400 MHz, DMSO-d_6) : δ = 0.80-0.92 (m, 3H, -CH_2-C*H*$_3$-), 1.16-1.67 (m, 15H, -CH-C*H*$_2$-C*H*$_2$-CH_3-, -CH_2-C*H*$_2$-C*H*$_2$-C*H*$_2$-C*H*$_2$-CH_2-, -C*H*$_3$-CH-), 2.84-2.99 (s, 12H, -N-C*H*$_3$, -N^+-C*H*$_3$), 2.99-3.13 (m, 2H, -NH-C*H*$_2$-CH_2-), 3.22-3.34 (m, 4H, -CH_2-C*H*$_2$-NH-, -S-C*H*$_2$-CH_2-), 4.60-4.74 (m, 2H, -(C=O)-C*H*-CH_3), 6.41-6.60 (m, 6H, benzene-*H*), 6.70-6.82 (s, 1H, -CH_2-N*H*-(C=O)-), 7.25-8.47 (m, 3H, benzene-*H*), 8.76-8.86 (s, 1H, -(C=O)-N*H*-CH_2-). ^{13}C NMR (100 MHz, DMSO-d_6): δ = 223.0, 169.1, 164.6, 136.3, 128.6, 97.7, 55.9, 50.2, 39.5, 36.0, 29.6, 29.0, 28.8, 26.2, 26.1, 21.5, 18.6, 18.2, 13.5 ppm; HR-MS (m/z) found 749.2867, calcd 749.2860 $[M + H]^+$.

Scheme S3. Synthesis of Rh-CTA.

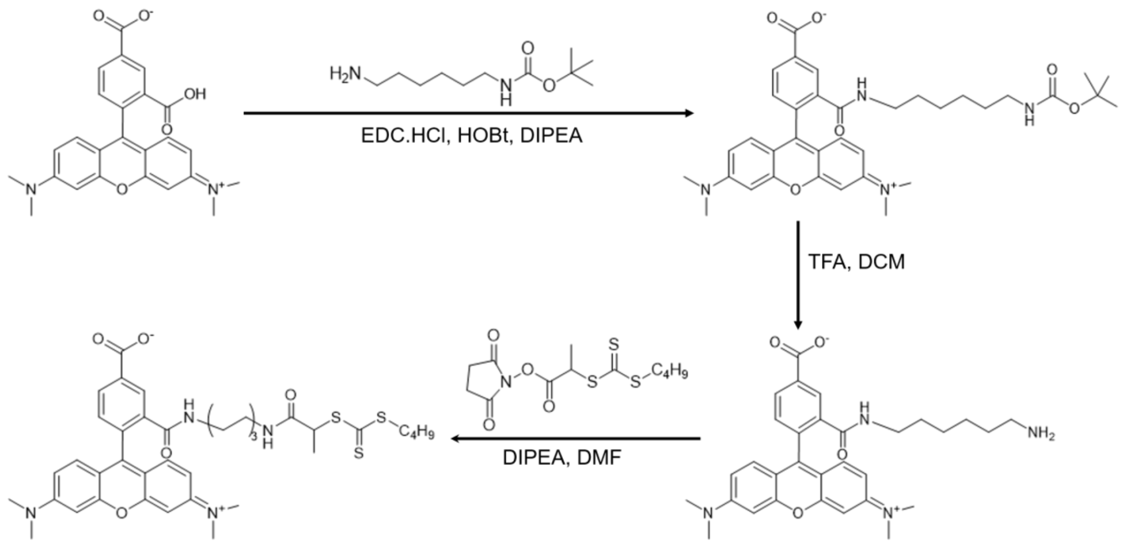

Syntheses of PDMA, PDMA-*b*-PAAc$_{20}$, PDMA-*b*-PTAc$_{20}$ (PT), PDMA-*b*-PMTAc$_{20}$ (PMT)

The typical procedure was as follows. For PDMA, a 100 mL ampoule was charged with DMA (5 mL, 48.4 mmol), 2-(((butylthio)carbonothiolyl)thio)propanoic acid (288 mg, 1.21 mmol), VA-044 (34.7 mg, 0.121 mmol) and a mixture of DMF and water (5 mL, v:v 1:4). The mixture was thoroughly degassed *via* 3 freeze-pump-thaw cycles, filled with nitrogen and then immersed in an oil bath at 70 °C for 2 h. The polymerization solution was diluted with DMF and water and directly used in the next reaction. The yellow polymer was analyzed by ^{1}H NMR spectroscopy and SEC (DMF + 5 mM NH_4BF_4 as eluent). The degree of polymerization (DP) of this PDMA macro-CTA was calculated to be 40 using ^{1}H NMR spectroscopy by multiplying the monomer conversion and the feeding degree of polymerization (DP). The syntheses of PDMA-*b*-PAAc$_{20}$, PDMA-*b*-PTAc$_{20}$ (PT) and PDMA-*b*-PMTAc$_{20}$ (PMT) were carried out according to the similar procedure for PDMA (Scheme S4). For example, the synthesis of PDMA$_{40}$-*b*-PAAc$_{20}$ (PA0)

was as follows. $PDMA_{40}$ (84.1 mg, 0.02 mmol), AAc (133.3 mg, 0.4 mmol), and VA-044 (0.88 mg, 0.003 mmol) were charged into the reaction vessel with DMF and water. The mixture was thoroughly degassed *via* 3 freeze-pump-thaw cycles, filled with nitrogen, and then immersed in an oil bath at 70 °C for 2 h with constant stirring. The ^{1}H NMR spectra and SEC traces of these polymers are presented in Figures S6 and S16-S19.

Scheme S4. Synthesis of PDMA-*b*-$PAAc_{20}$, PDMA-*b*-$PTAc_{20}$ (PT), PDMA-*b*-$PMTAc_{20}$ (PMT).

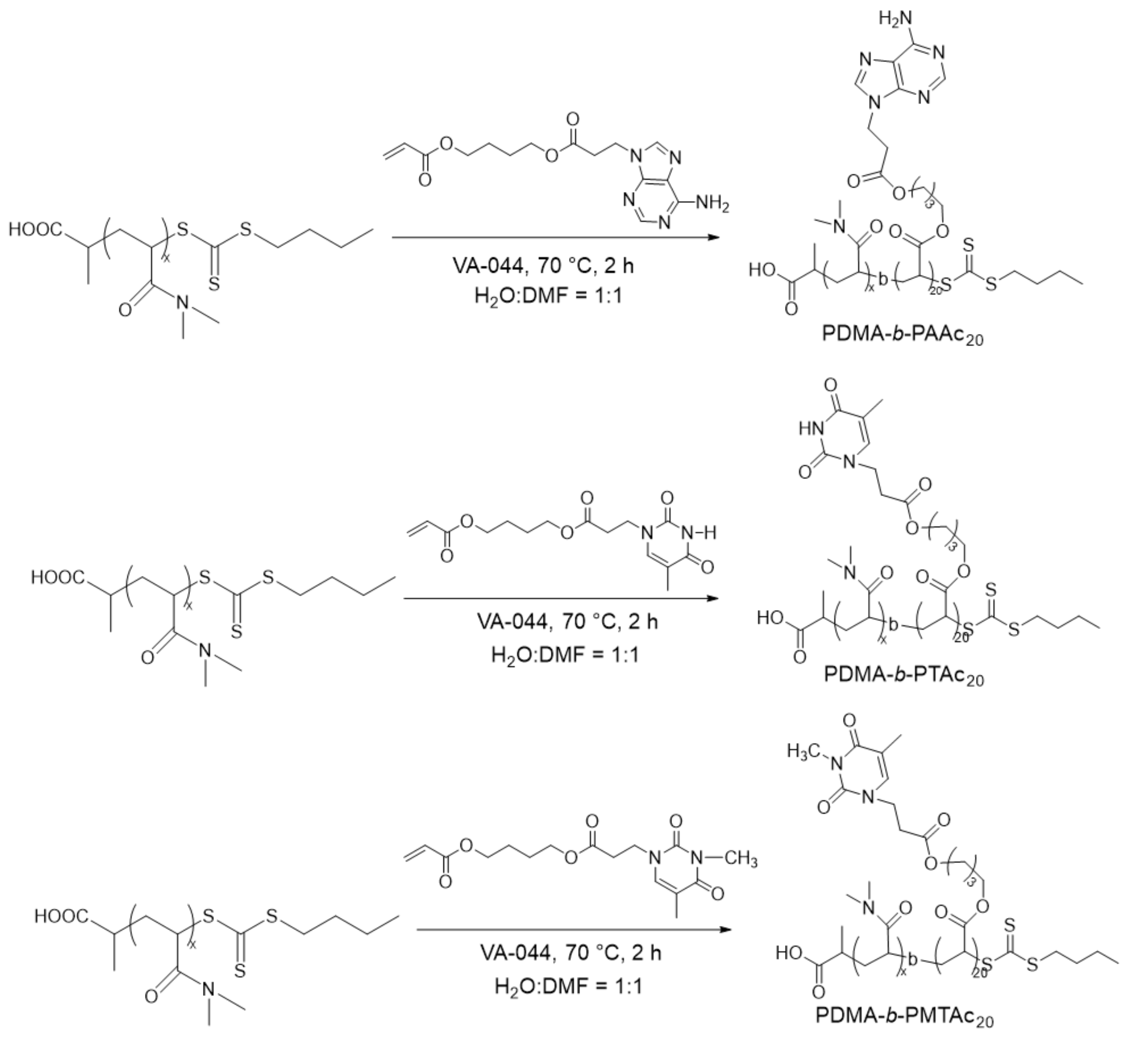

Synthesis of PDMA$_{40}$-*b*-PAAc$_{20}$-*b*-PMCAcs

Taking PDMA$_{40}$-*b*-PAAc$_{20}$-*b*-PMCAc$_{20}$ (PA) as an example, PDMA$_{40}$-*b*-PTAc$_{20}$ (108.7 mg, 0.01 mmol), MCAc (54.9 mg, 0.2 mmol), and AIBN (0.246 mg, 0.0015 mmol) were dissolved in DMAc (654 μL). The mixture was thoroughly degassed *via* 3 freeze-pump-thaw cycles, filled with nitrogen and then immersed in an oil bath at 80 °C for 2 h (Scheme S5). An aliquot of the crude product was taken and analyzed by ^{1}H NMR spectroscopy to calculate the conversion. The degree of polymerization (DP) of obtained triblock copolymers was calculated using the conversion from ^{1}H NMR spectroscopy. The residual solution was then precipitated three times from the mixture of diethyl ether and methanol (v:v 3:1). The polymer was dried in a vacuum oven overnight at room temperature and analyzed by ^{1}H NMR spectroscopy and SEC (DMF + 5 mM NH_4BF_4 as eluent) (Figure S7). The synthetic procedures of PDMA$_{40}$-*b*-PAAc$_{20}$-*b*-PMCAc$_{10/30}$ were the same as the synthesis of PDMA$_{40}$-*b*-PAAc$_{20}$-*b*-PMCAc$_{20}$ (PA) (Figures S11-S12).

Scheme S5. Synthesis of PDMA$_{40}$-*b*-PAAc$_{20}$-*b*-PMCAcs.

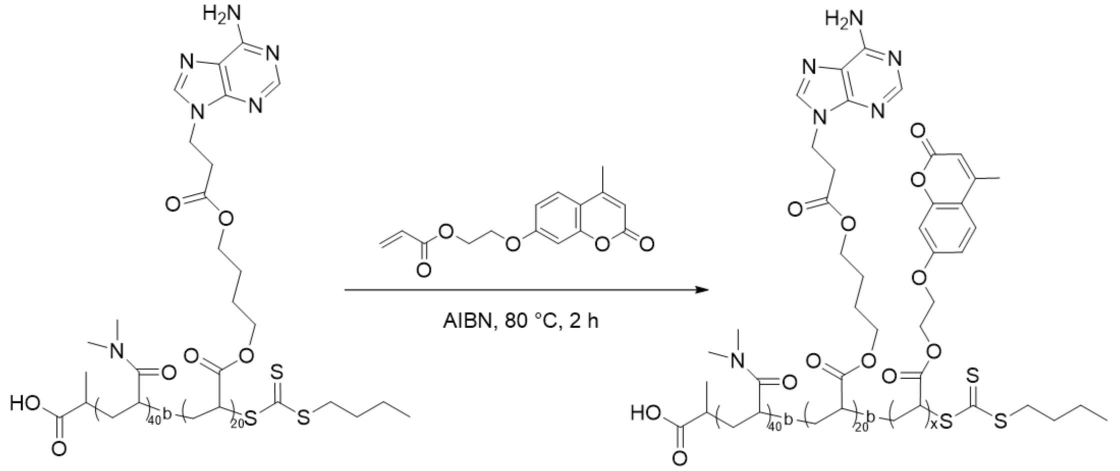

Synthesis of $PAMPS_{40}$-*b*-$PTAc_{20}$ (PT4) and $PTMPA_{40}$-*b*-$PTAc_{20}$ (PT5)

$PAMPS_{40}$-*b*-$PTAc_{20}$ copolymers were prepared by using reversible addition-fragmentation chain transfer (RAFT) polymerization. A typical synthetic procedure is as follows. For $PAMPS_{40}$ Macro-CTA, an ampule was charged with AMPS (124.4 mg, 0.60 mmol), 2-(((butylthio)carbonothiolyl)thio)propanoic acid (3.57 mg, 0.015 mmol), VA-044 (0.430 mg, 0.0015 mmol), and a mixture of DMF and water (515 µL, v:v 1:4). The mixture was thoroughly degassed *via* 3 freeze-pump-thaw cycles, filled with nitrogen, and then immersed in an oil bath at 70 °C for 2 h with constant stirring (Scheme S6). The polymerization solution was then exposed to air to quench the residual radicals. The polymerization mixture was taken and analyzed by ^1H NMR spectroscopy and SEC to calculate the conversion. Due to the high conversion of the AMPS monomer, the polymerization solution was directly used for chain extension without further purifications. Then, TAc (91.9 mg, 0.28 mmol), VA-044 (0.61 mg, 2.13×10^{-3} mmol), and a mixture of DMF and water (854 µL, v:v 1:1) were added into the ampule. The mixture was thoroughly degassed *via* 3 freeze-pump-thaw cycles, filled with nitrogen, and then immersed in an oil bath at 70 °C for 2 h. The polymerization solution was exposed to air to quench the residual radicals. An aliquot of the crude product was taken and analyzed by ^1H NMR spectroscopy to calculate the conversion. The residual solution was precipitated three times from cold diethyl ether. The polymer was dried in a vacuum oven overnight at room temperature and analyzed by ^1H NMR spectroscopy and DMF SEC (Figure S22). The synthetic procedure of $PTMPA_{40}$-*b*-$PTAc_{20}$ (PT5) (Figure S23) was same as the synthesis of $PAMPS_{40}$-*b*-$PTAc_{20}$

(PT4).

Scheme S6. Synthesis of $PAMPS_{40}$-*b*-$PTAc_{20}$ (PT4) and $PTMPA_{40}$-*b*-$PTAc_{20}$ (PT5).

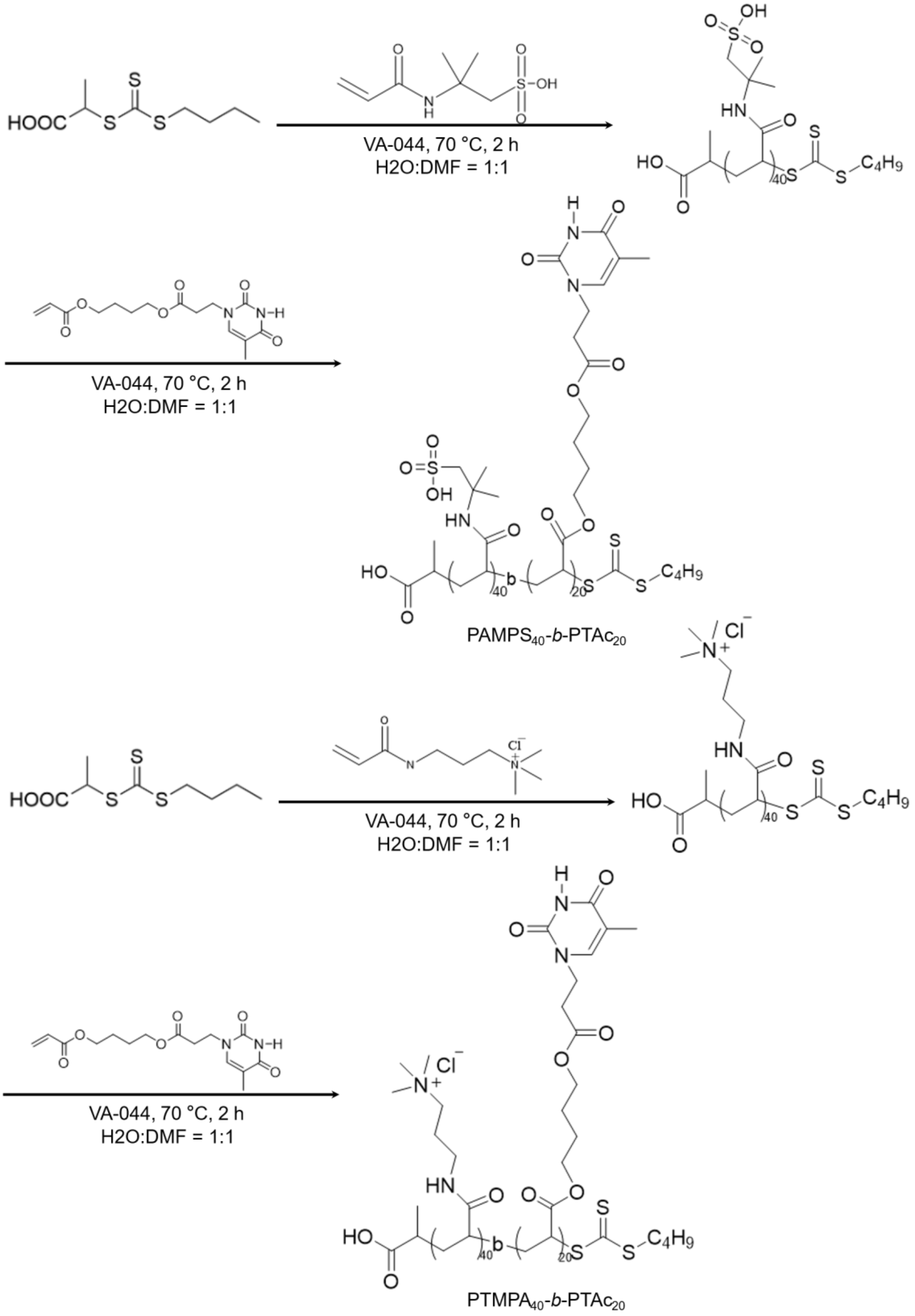

Synthesis of biotin-attached PDMA$_{40}$-*b*-PTAc$_{20}$ (PT1b) and rhodamine-modified PDMA$_{40}$-*b*-PTAc$_{20}$ (PT1Rh)

A typical synthetic procedure is as follows. For biotin-attached PDMA$_{40}$, an ampule was charged with DMA (79.3 mg, 0.8 mmol), biotin-CTA (10.1 mg, 0.01 mmol), VA-044 (0.287 mg, 0.001 mmol), and a mixture of DMF and water (360 µL, v:v 1:1). The mixture was thoroughly degassed *via* 3 freeze-pump-thaw cycles, filled with nitrogen, and then immersed in an oil bath at 70 °C for 2 h with constant stirring. The polymerization solution was exposed to air to quench the residual radicals. The polymerization mixture was taken and analyzed by ^{1}H NMR spectroscopy and SEC to calculate the conversion. Due to the high conversion of the DMA monomer, the polymerization solution was directly used for chain extension without further purifications. Then, TAc (60.5 mg, 0.187 mmol), VA-044 (0.40 mg, 1.40×10^{-3} mmol), and a mixture of DMF and water (411 µL, v:v 1:1) were added into the ampule. The mixture was thoroughly degassed *via* 3 freeze-pump-thaw cycles, filled with nitrogen, and then immersed in an oil bath at 70 °C for 2 h (Scheme S7). The polymerization solution was exposed to air to quench the residual radicals. An aliquot of the crude product was taken and analyzed by ^{1}H NMR spectroscopy to calculate the conversion. The residual solution was precipitated three times from cold diethyl ether. The polymer was dried in a vacuum oven overnight at room temperature and analyzed by ^{1}H NMR spectroscopy and DMF SEC (Figure S25). The synthetic strategy for PT1Rh (Figure S28) is similar to that of PT1b, but differs in the monomer polymerization sequence. Specifically, the preparation of PT1Rh involved the construction of a precursor through polymerization

of TAc monomer with Rh-CTA, followed by a second-stage chain-extension polymerization with DMA monomer. As a result, the fluorescent group of rhodamine was attached at the hydrophobic end.

Scheme S7. Synthesis of biotin-attached $PDMA_{40}$-*b*-$PTAc_{20}$ (PT1b) and rhodamine-modified $PDMA_{40}$-*b*-$PTAc_{20}$ (PT1Rh).

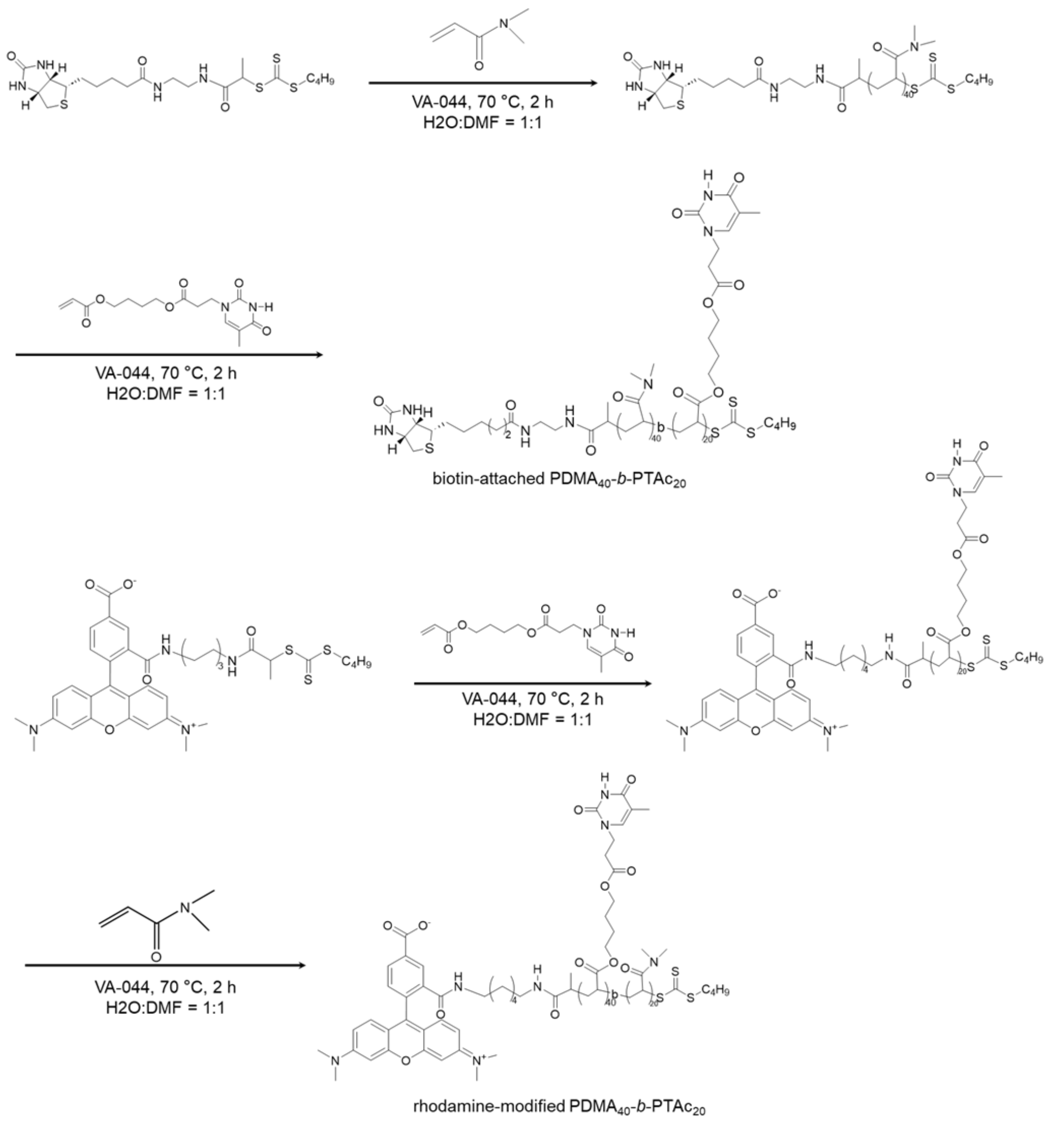

Crosslinking and purification of self-assembled polymer nanoparticles

The synthesized triblock copolymer $PDMA_{40}$-*b*-$PAAc_{20}$-*b*-PMCAc was dissolved in DMF at *ca.* 8 mg mL^{-1}. Under vigorous stirring, deionized water was slowly dropped into the solution at a rate of 10 μL min^{-1} for solvent switch, successfully yielding polymeric micelles. The resulting solution appeared transparent with a pale blue tint and exhibited a clearly visible light path due to the Tyndall effect when irradiated with a laser pointer. Subsequently, the micelles were cross-linked by irradiation with 302 nm ultraviolet (UV) light. The core-crosslinked polymer nanoparticles were purified and separated by using preparative SEC. Finally, the obtained solution was dialyzed (MWCO 3500 Da) against deionized water, during which the deionized water was changed every 4 h for at least 2 days. Following concentration via freeze-drying, the final aqueous polymer solution was obtained. The nanostructure was further characterized by using DLS, TEM.

Interactions between McA nanostructure and MTs

The interaction between McA nanostructure and distinct MTs follows the similar procedures. Taking the synthesis of McA-PT1 as an example, the solutions of MT1 were prepared by directly dissolving PT1 in water at *ca.* 10 mg mL^{-1}. The aqueous solution of McA was concentrated at a concentration of 0.8 mg mL^{-1}. The McA and MT solutions were mixed at an A:T molar ratio of 1:1 and stirred continuously for 2 hours to allow efficient interaction. The attained combined nanostructure was analyzed by using DLS and TEM.

Supplementary Figures

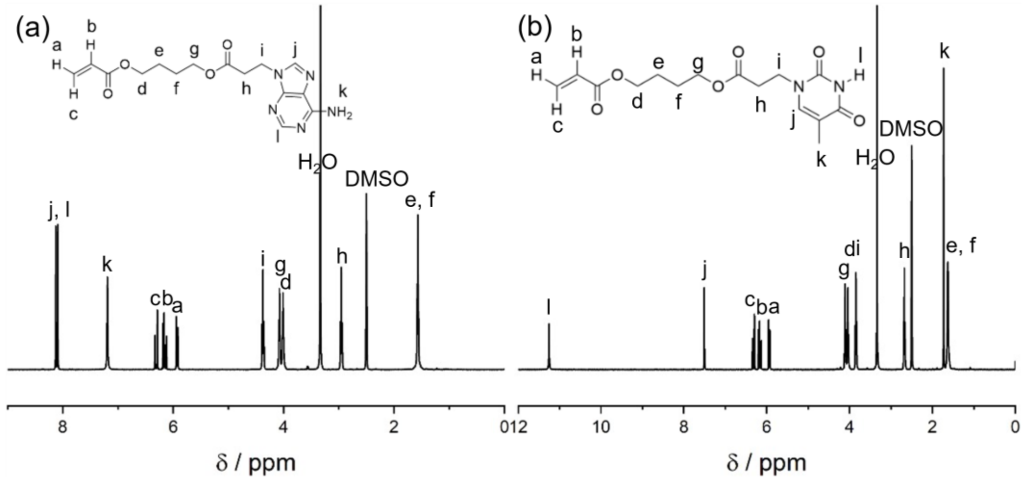

Figure S1. ^{1}H NMR spectra of (a) 4-((3-(adenin-9-yl)propanoyl)oxy)butyl acrylate (AAc) and (b) 4-((3-(thymin-l-yl)propanoyl)oxy)butyl acrylate (TAc) in DMSO-d_6.

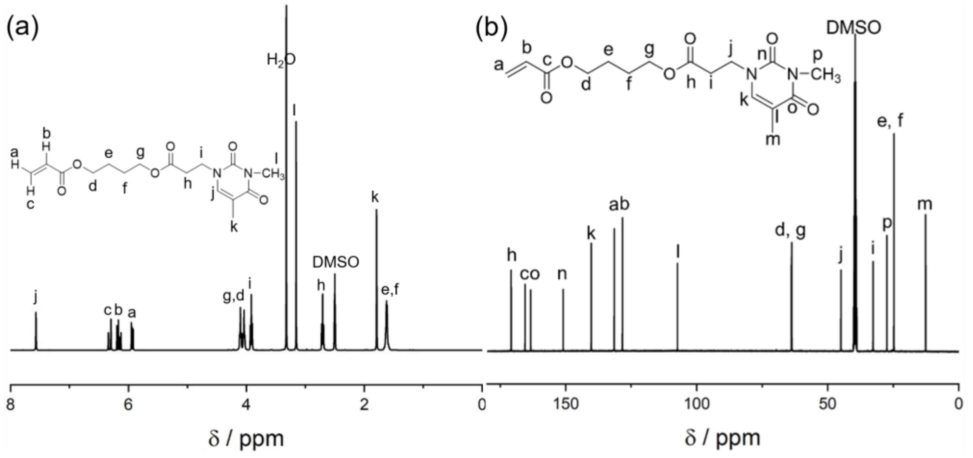

Figure S2. Assigned (a) ^{1}H and (b) ^{13}C NMR spectra of 4-((3-(methylthymin-1-yl)-propanoyl)oxy)butyl acrylate (MTAc) in DMSO-d_6.

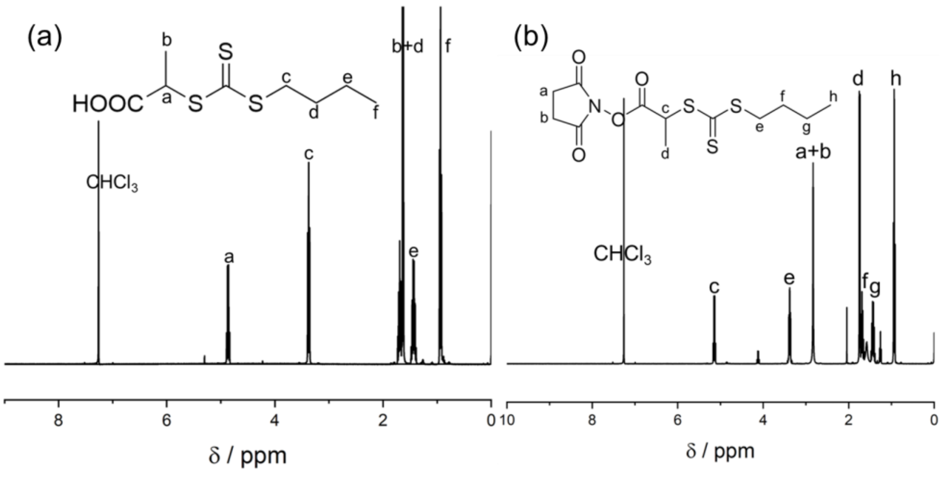

Figure S3. ^{1}H NMR spectra of (a) CTA and (b) CTA-NHS in $CDCl_3$.

Scheme S7. Synthesis of 2-[(4-methyl-2-oxo-2H-7-chromenyl)oxy]ethyl acrylate (MCAc).

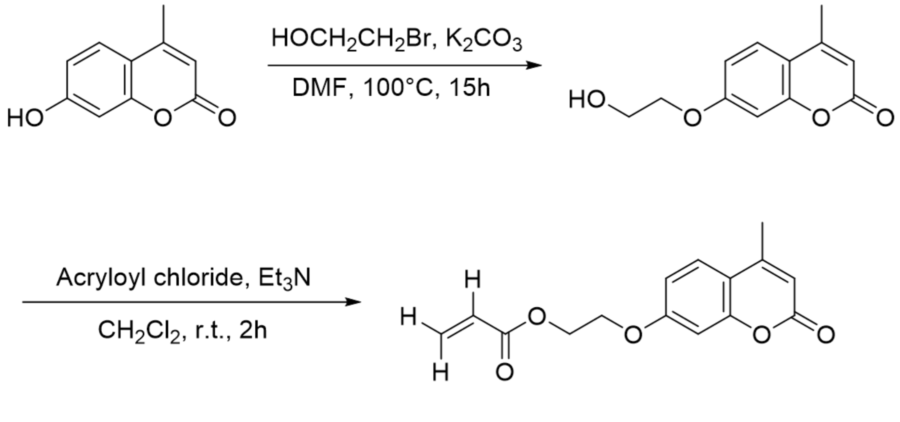

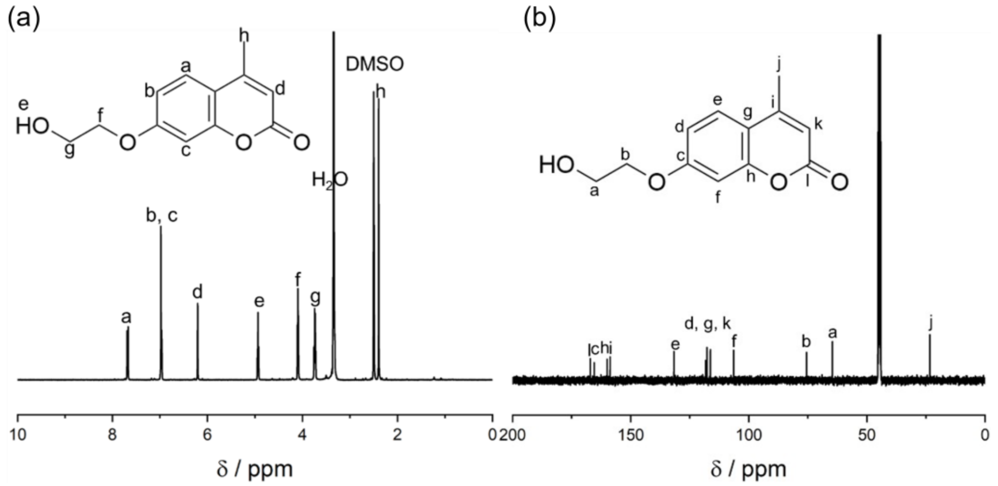

Figure S4. Assigned (a) ^{1}H and (b) ^{13}C NMR spectra of 2[(4-methyl-2-oxo-2H-7-chromenyl)oxy]ethanol in DMSO-d_6.

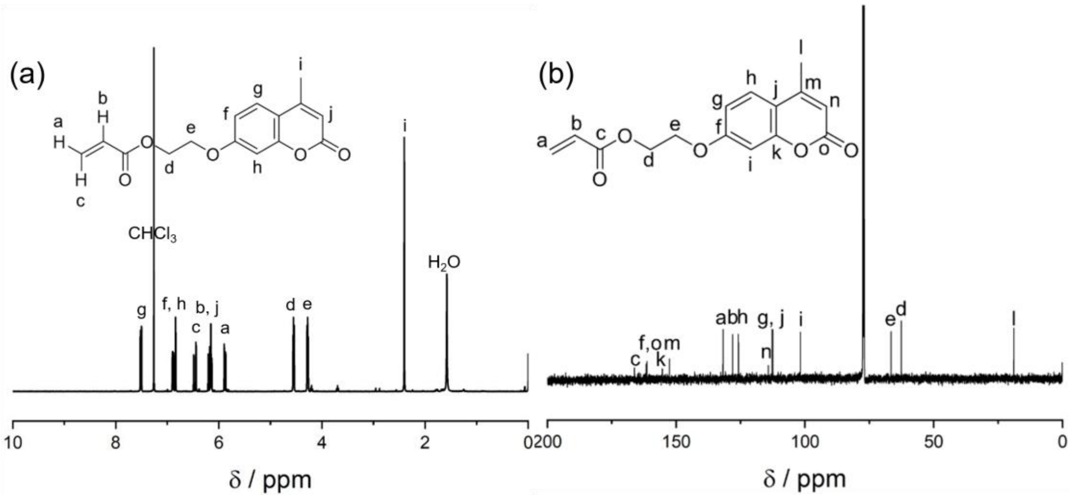

Figure S5. Assigned (a) ^{1}H and (b) ^{13}C NMR spectra of 2-[(4-methyl-2-oxo-2H-7-chromenyl)oxy]ethyl acrylate (MCAc) in $CDCl_3$.

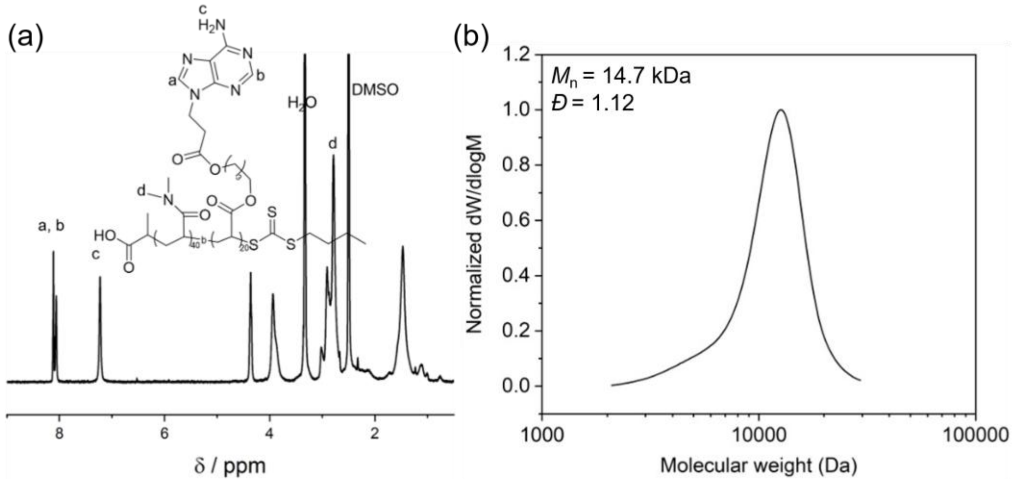

Figure S6. (a) ^{1}H NMR spectrum (in DMSO-d_6) and (b) DMF SEC trace of PDMA$_{40}$-*b*-PAAc$_{20}$ (PA0).

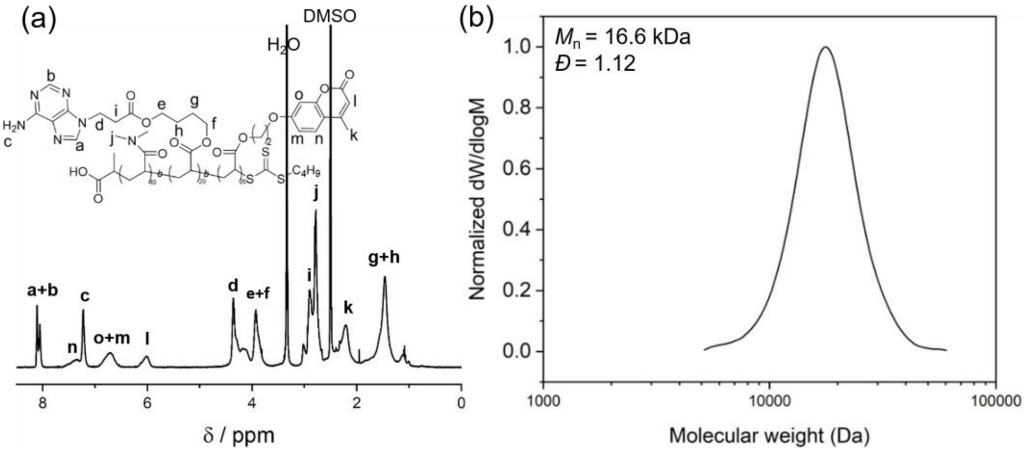

Figure S7. (a) ^{1}H NMR spectrum (in DMSO-d_6) and (b) SEC trace of PDMA$_{40}$-*b*-PAAc$_{20}$-*b*-PMCAc$_{20}$ (PA).

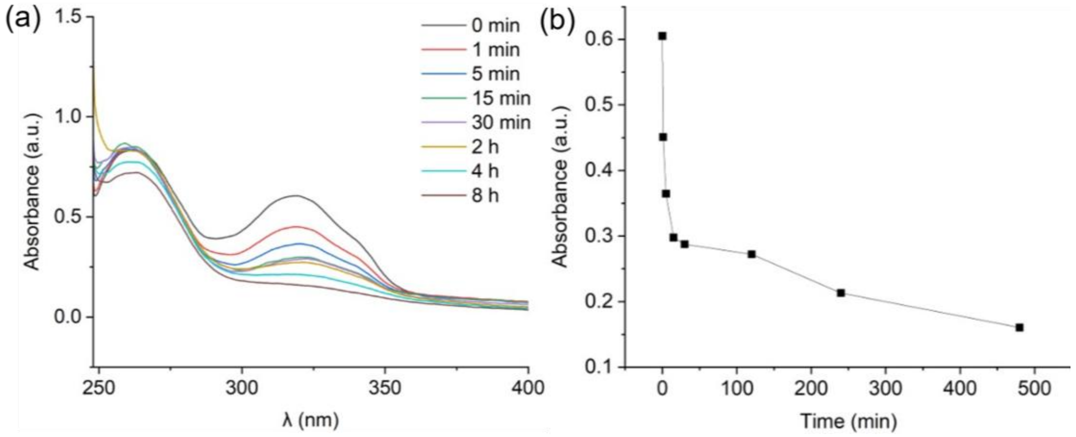

Figure S8. (a) The crosslinking kinetics of MA were monitored by using UV-vis and (b) the UV absorbance at 319 nm.

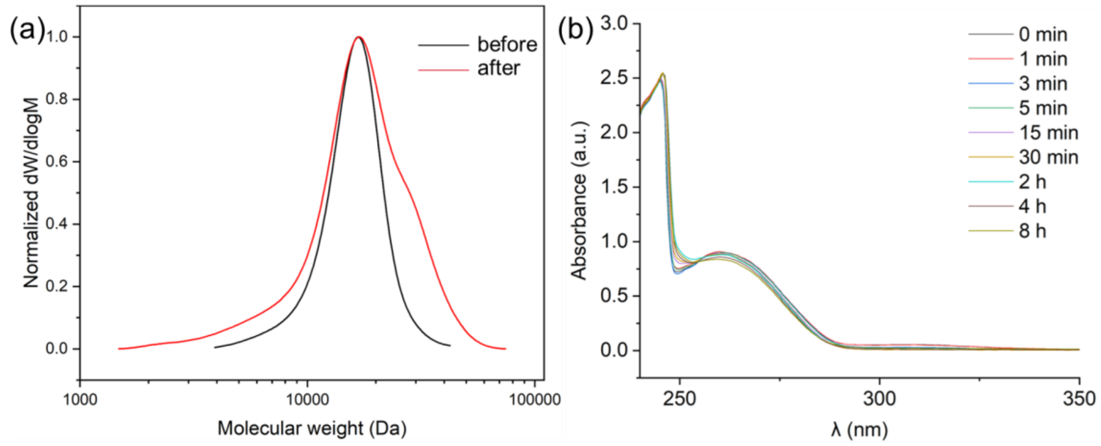

Figure S9. (a) SEC traces of the $PDMA_{40}$-*b*-$PAAc_{20}$ before and after crosslinking. (b) UV-vis variation of $PDMA_{40}$-*b*-$PAAc_{20}$ under ultraviolet irradiation.

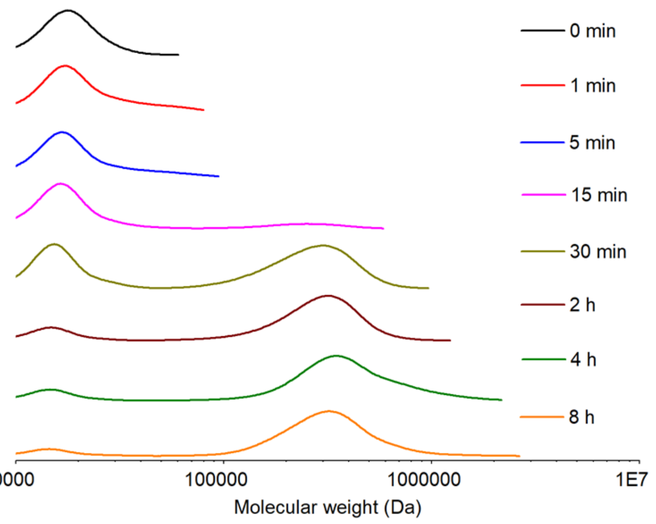

Figure S10. The crosslinking kinetics of MA were monitored by using SEC analyses.

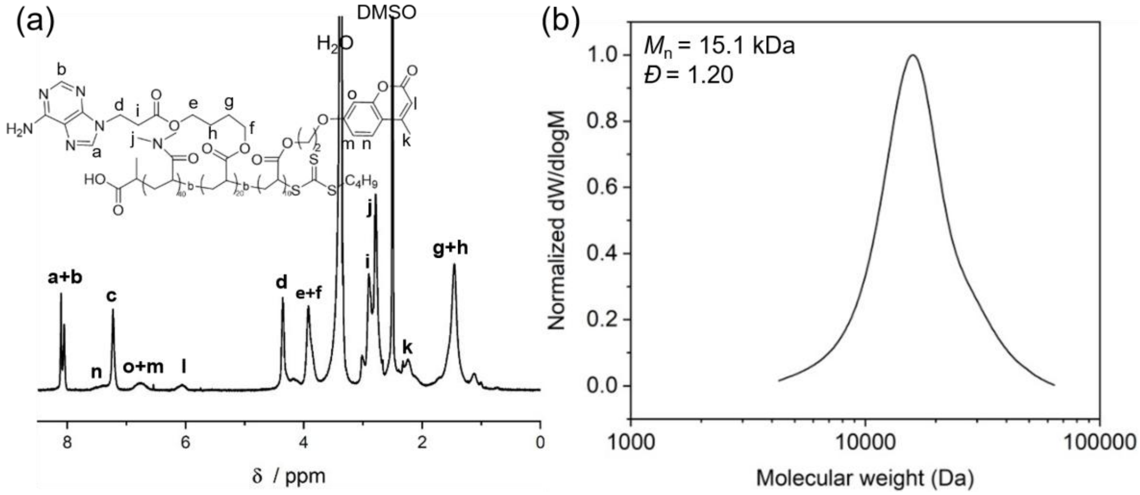

Figure S11. (a) ^{1}H NMR spectrum (in DMSO-d_6) and (b) SEC trace of $PDMA_{40}$-*b*-$PAAc_{20}$-*b*-$PMCAc_{10}$.

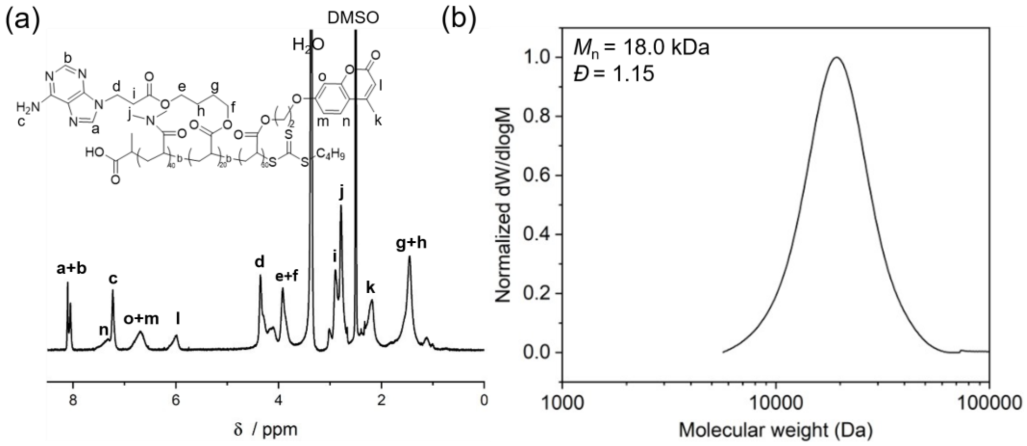

Figure S12. (a) ^{1}H NMR spectrum (in DMSO-d_6) and (b) SEC trace of PDMA$_{40}$-*b*-PAAc$_{20}$-*b*-PMCAc$_{30}$.

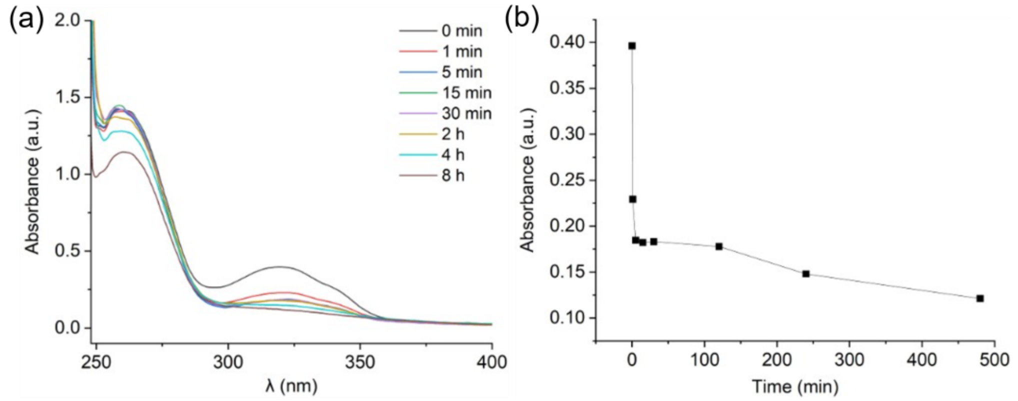

Figure S13. (a) The crosslinking kinetics of the assembly of PDMA$_{40}$-*b*-PAAc$_{20}$-*b*-PMCA$_{10}$ were monitored by using UV-vis and (b) the UV absorbance at 319 nm.

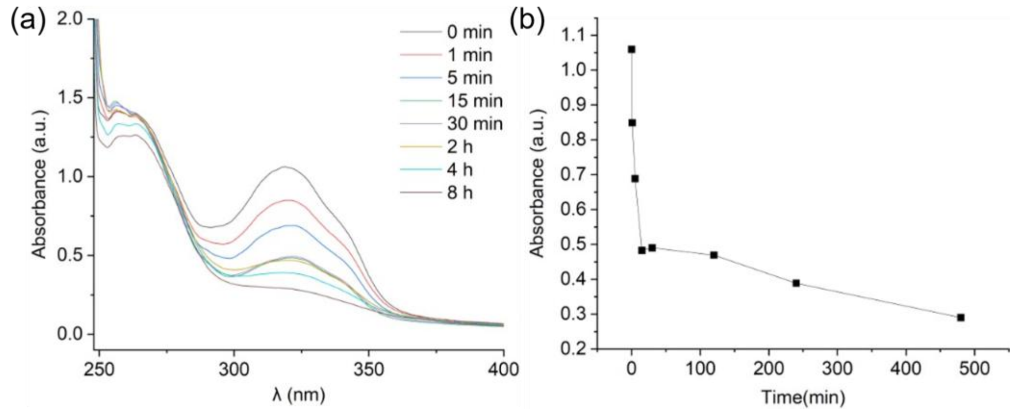

Figure S14. (a) The crosslinking kinetics of the assembly of PDMA$_{40}$-*b*-PAAc$_{20}$-*b*-PMCA$_{30}$ were monitored by using UV-vis and (b) the UV absorbance at 319 nm.

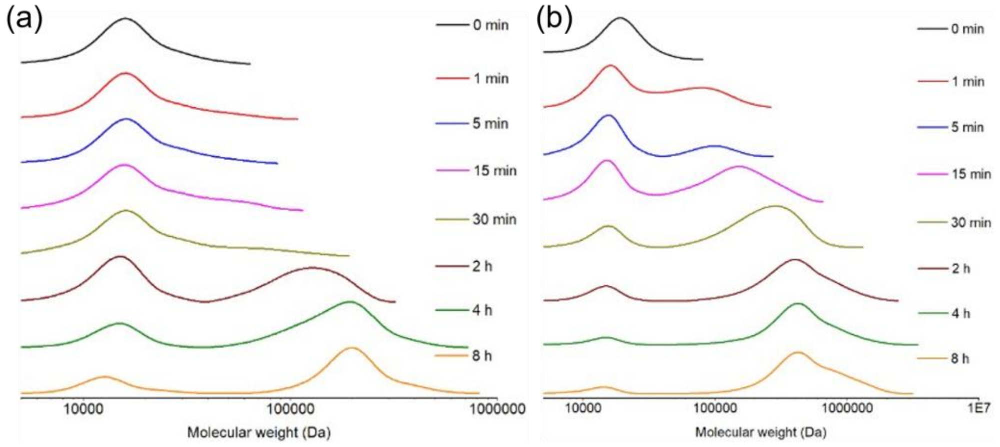

Figure S15. The crosslinking kinetics of the assembly of (a) $PDMA_{40}$-*b*-$PAAc_{20}$-*b*-$PMCA_{10}$ (b) $PDMA_{40}$-*b*-$PAAc_{20}$-*b*-$PMCA_{30}$ were monitored by using SEC analyses.

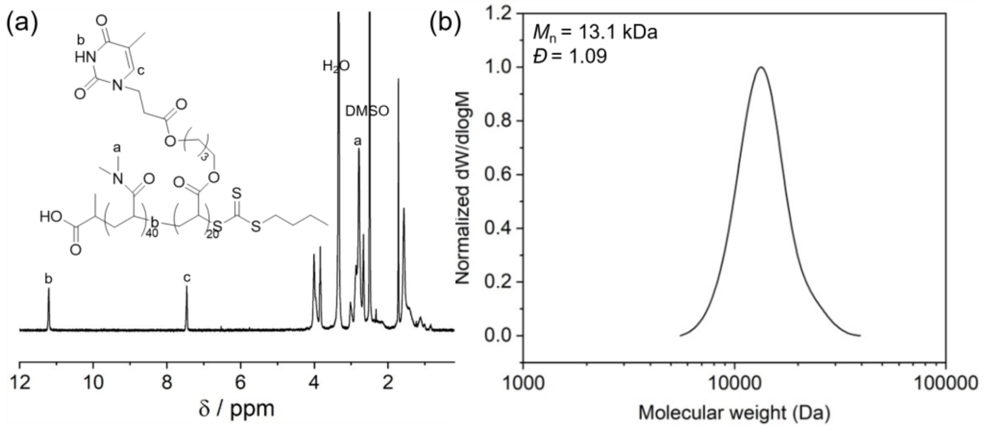

Figure S16. (a) ^1H NMR spectrum (in DMSO-d_6) and (b) SEC trace of $PDMA_{40}$-*b*-$PTAc_{20}$ (PT1).

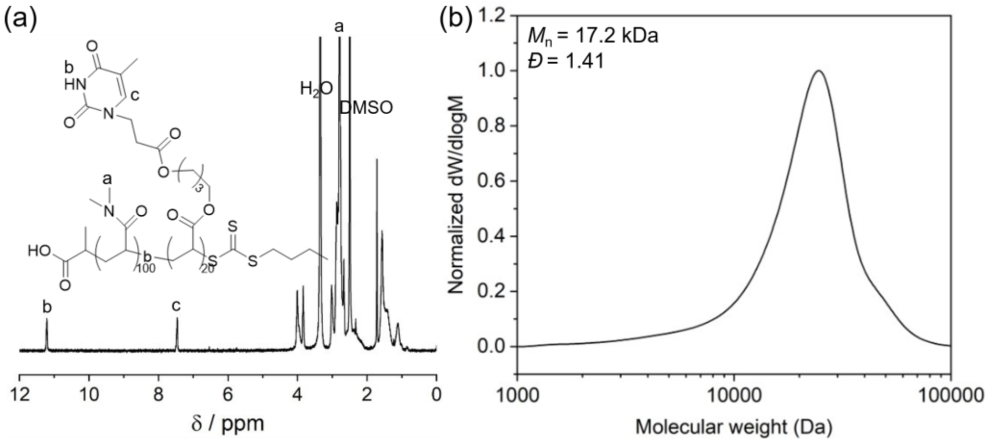

Figure S17. (a) ^1H NMR spectrum (in DMSO-d_6) and (b) SEC trace of $PDMA_{100}$-*b*-$PTAc_{20}$ (PT2).

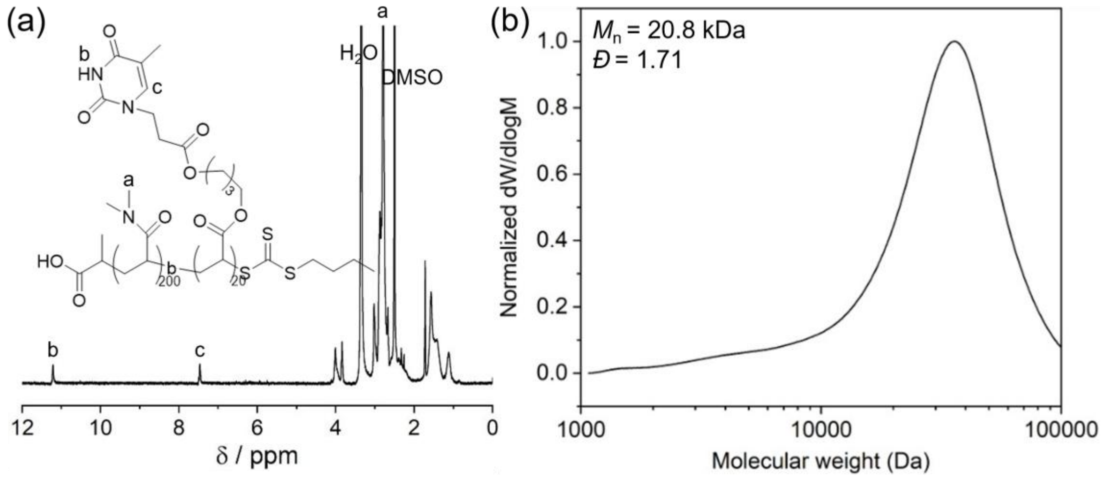

Figure S18. (a) ^{1}H NMR spectrum (in DMSO-d_6) and (b) SEC trace of PDMA$_{200}$-*b*-PTAc$_{20}$ (PT3).

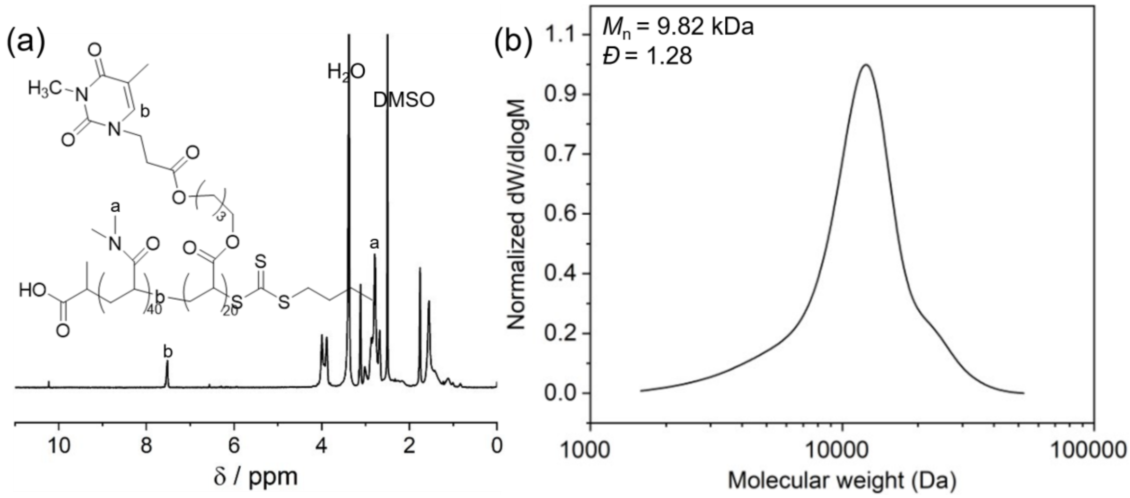

Figure S19. (a) ^{1}H NMR spectrum (in DMSO-d_6) and (b) SEC trace of PDMA$_{40}$-*b*-PMTAc$_{20}$ (PMT).

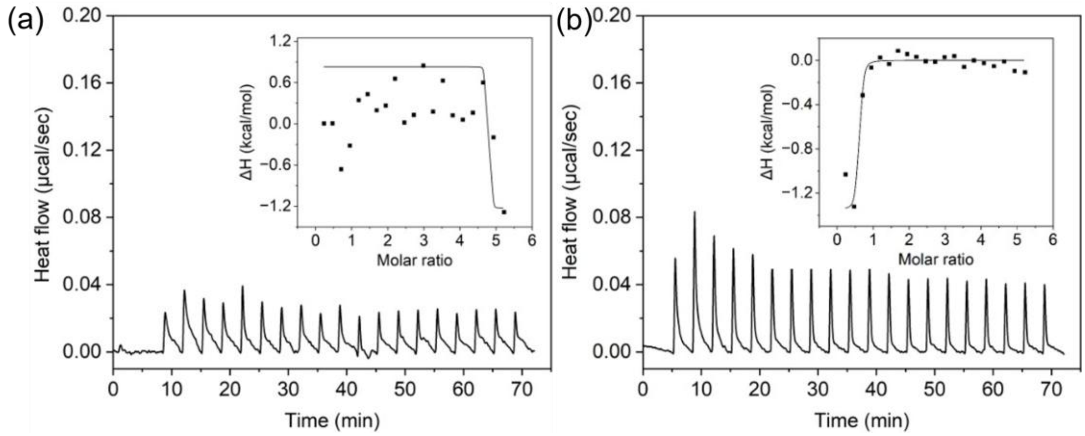

Figure S20. ITC analyses of the interaction between McA with (a) PA0, (b) PMT.

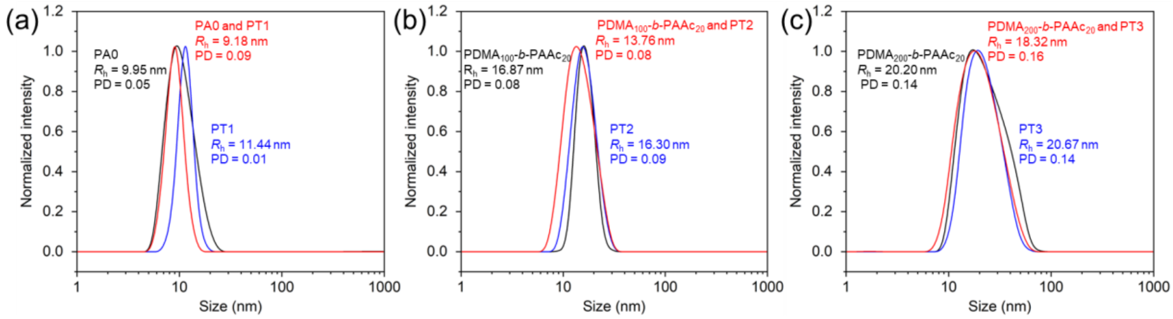

Figure S21. DLS analyses of the mixing of non-crosslinked (a) PA0 and PT1, (b) PDMA$_{100}$-*b*-PAAc$_{20}$ and PT2, (c) PDMA$_{200}$-*b*-PAAc$_{20}$ and PT3.

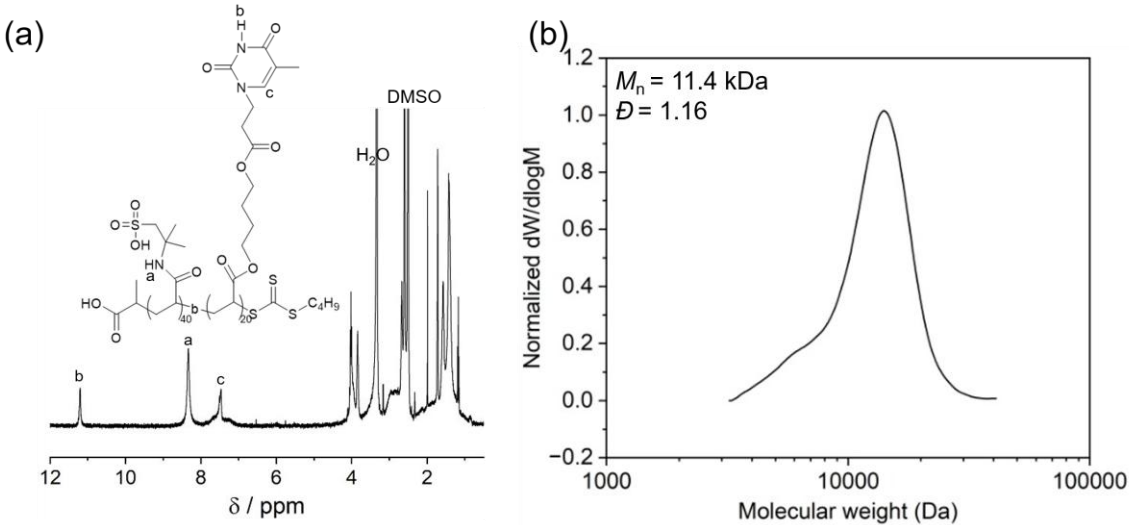

Figure S22. (a) ^{1}H NMR spectrum (in DMSO-d_6) and (b) SEC trace of PAMPS$_{40}$-*b*-PTAc$_{20}$ (PT4).

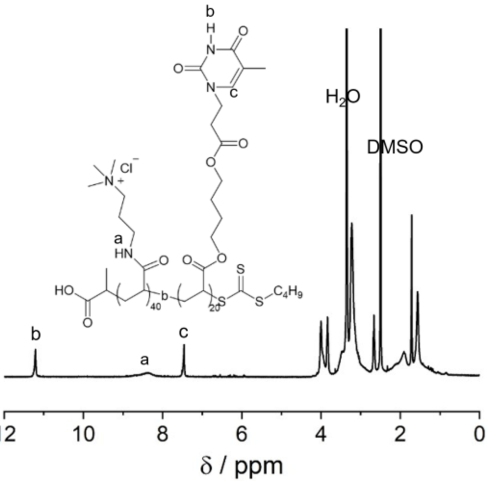

Figure S23. ^{1}H NMR spectrum of PTMPA$_{40}$-*b*-PTAc$_{20}$ in DMSO-d_6.

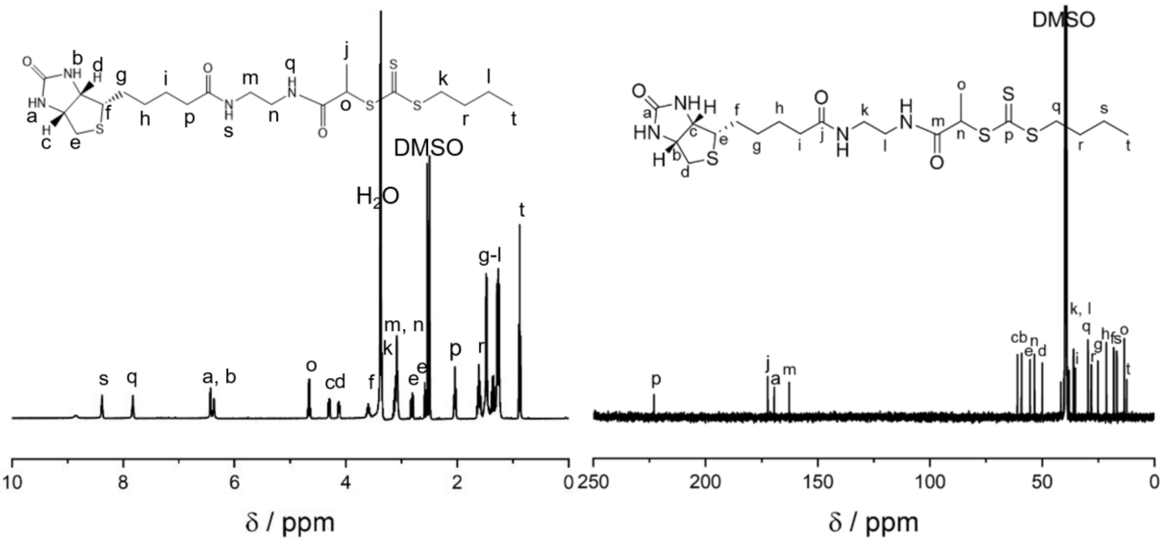

Figure S24. (a) ^{1}H and (b) ^{13}C NMR spectra of biotin-CTA in DMSO-d_6.

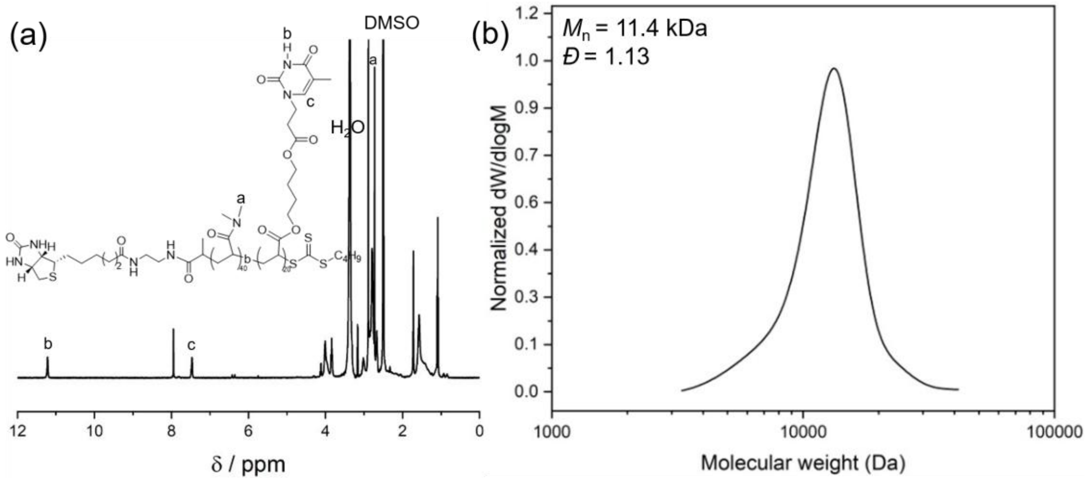

Figure S25. (a) ^{1}H NMR spectrum (in DMSO-d_6) and (b) SEC trace of biotin-attached PDMA$_{40}$-*b*-PTAc$_{20}$ (PT1b).

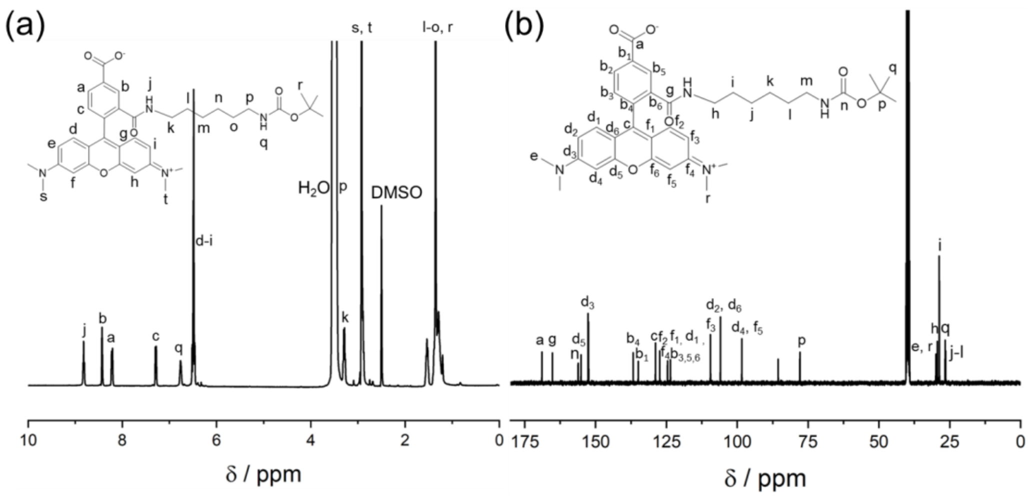

Figure S26. Assigned (a) ^{1}H and (b) ^{13}C NMR spectra of Rh-BocNH$_2$ in DMSO-d_6.

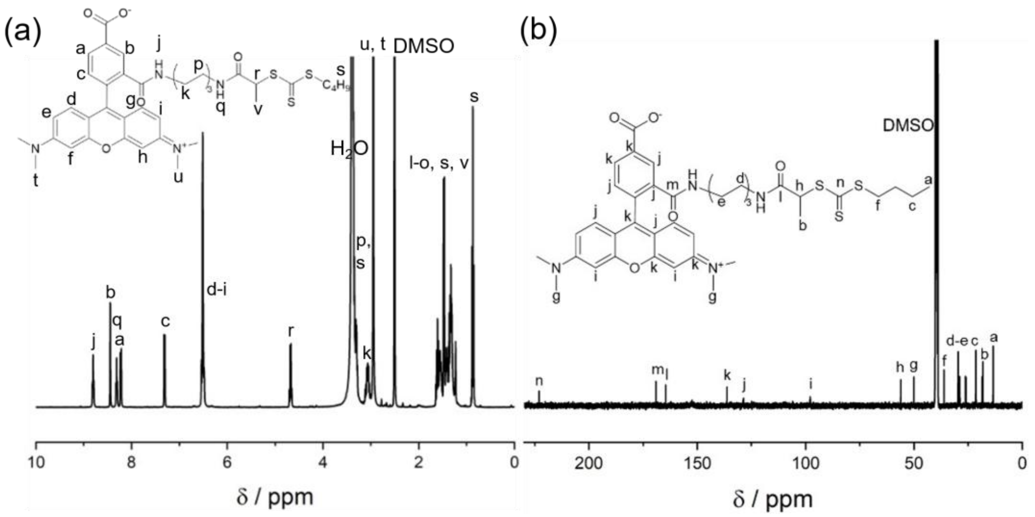

Figure S27. (a) ^1H and (b) ^{13}C NMR spectra of Rh-CTA in DMSO-d_6.

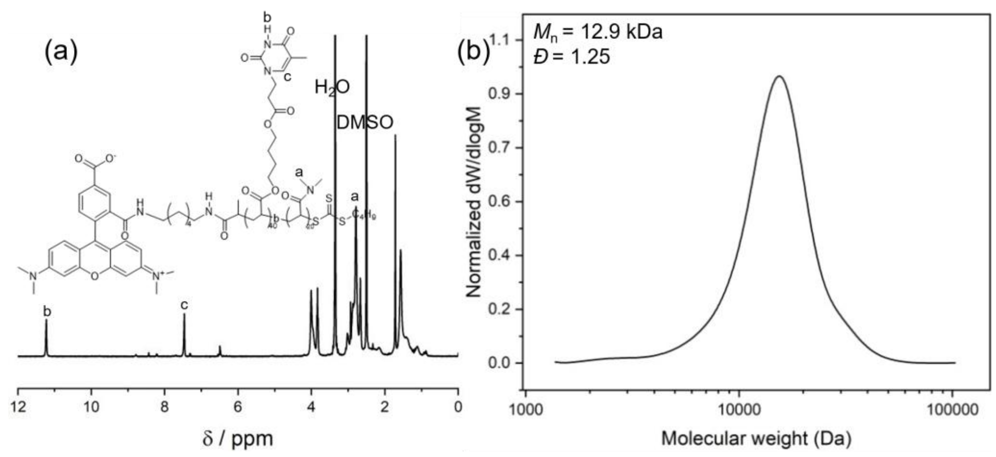

Figure S28. (a) ^1H NMR spectrum (in DMSO-d_6) and (b) SEC trace of rhodamine-modified PDMA$_{40}$-*b*-PTAc$_{20}$ (PT1Rh).

References

1. Z. Hua, A. Pitto-Barry, Y. Kang, N. Kirby, T. R. Wilks and R. K. O'Reilly, Micellar nanoparticles with tuneable morphologies through interactions between nucleobase-containing synthetic polymers in aqueous solution, *Polym. Chem*., 2016, **7**, 4254-4262.
2. T. Lueckerath, T. Strauch, K. Koynov, C. Barner-Kowollik, D. Y. W. Ng and T. Weil, DNA–Polymer Conjugates by Photoinduced RAFT Polymerization, *Biomacromolecules*, 2018, **20**, 212-221.
3. S. Cheng, M. Zhang, N. Dixit, R. B. Moore and T. E. Long, Nucleobase Self-Assembly in Supramolecular Adhesives, *Macromolecules*, 2012, **45**, 805-812.
4. K. H. Shaughnessy, P. Kim and J. F. Hartwig, A Fluorescence-Based Assay for High-Throughput Screening of Coupling Reactions. Application to Heck Chemistry, *J. Am. Chem. Soc*., 1999, **121**, 2123-2132.
